# Research progress on immobilized penicillin G acylase and industrial applications

**DOI:** 10.1039/d5ra09771a

**Published:** 2026-07-02

**Authors:** Pen Jin, Hongyi Tu, Nan Wang, Fengying Li, Xiaodong Wei, Zhenbin Chen

**Affiliations:** a College of Materials Science and Engineering, Lanzhou University of Technology Lanzhou 730050 China zhenbinchen@163.com; b State Key Laboratory of Gansu Advanced Non-ferrous Metal Materials, Lanzhou University of Technology Lanzhou 730050 China; c School of Chemical Engineering, Northwest Minzu University Lanzhou 730030 China

## Abstract

Immobilized penicillin G acylase (PGA) is a critical industrial biocatalyst for converting penicillin G into 6-aminopenicillanic acid (6-APA), a key intermediate in the synthesis of β-lactam antibiotics. Although various microorganisms can produce penicillin acylases, free PGA is highly susceptible to structural instability and environmental factors such as temperature and pH fluctuations, leading to a significant activity loss. Furthermore, the separation of free enzymes from reaction systems remains technically challenging, increasing operational complexity and cost. To overcome these limitations, immobilization strategies have been extensively explored. Immobilized PGA exhibits superior stability, reusability, and operational robustness, which are highly desirable for industrial applications. These advantages also lead to improved product purity, reduced contamination risks, and economic benefits. However, immobilization does not necessarily improve intrinsic catalytic efficiency, instead, it often involves a trade-off between catalytic activity and stability due to conformational constraints and mass transfer limitations. Although improved catalytic performance is sometimes reported, such enhancement generally reflects apparent activity rather than intrinsic catalytic efficiency. This review systematically summarizes the origins, classification, structure, and catalytic mechanisms of PGA. It also critically evaluates recent progress in immobilization methods, carrier materials, reaction media, enzyme activity regeneration, and reactor design, providing insights into future challenges and opportunities in this field.

## Abstract 2

This review examines PGA immobilization for 6-APA production, addressing the trade-off between activity and stability caused by diffusion constraints. It highlights scalable designs and stimuli-responsive systems as key future solutions.

## Graphical abstract

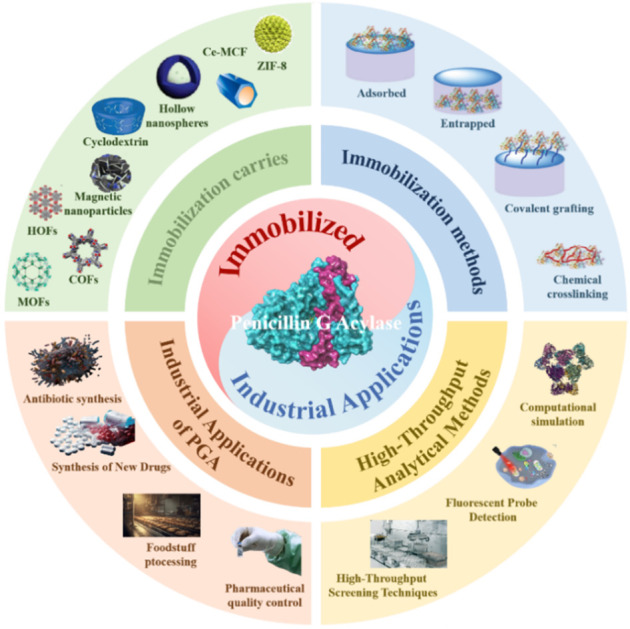

## 1 Introduction

Since Alexander Fleming's discovery of penicillin in 1932, penicillin-derived pharmaceuticals have been widely adopted in medical practice.^1^ As a member of the β-lactam antibiotics (BAs), penicillin has become a cornerstone of antibacterial chemotherapy due to its potent bactericidal activity, broad therapeutic scope, low toxicity, and high clinical efficacy.^2,3^ Its mechanism involves the inhibition of bacterial cell wall synthesis by binding to penicillin-binding proteins (PBPs) *via* its β-lactam ring.^3^ Currently, most natural penicillins serve as precursors for semi-synthetic penicillins and cephalosporins, with only a small fraction used directly in clinical treatment.^4^ A diverse range of semi-synthetic penicillins can be synthesized by modifying the side-chain structure of natural penicillins. These derivatives offer superior clinical efficacy, broader antimicrobial spectra, reduced toxicity, and enhanced bactericidal activity compared to their natural counterparts.^5^

**Fig. 1:**
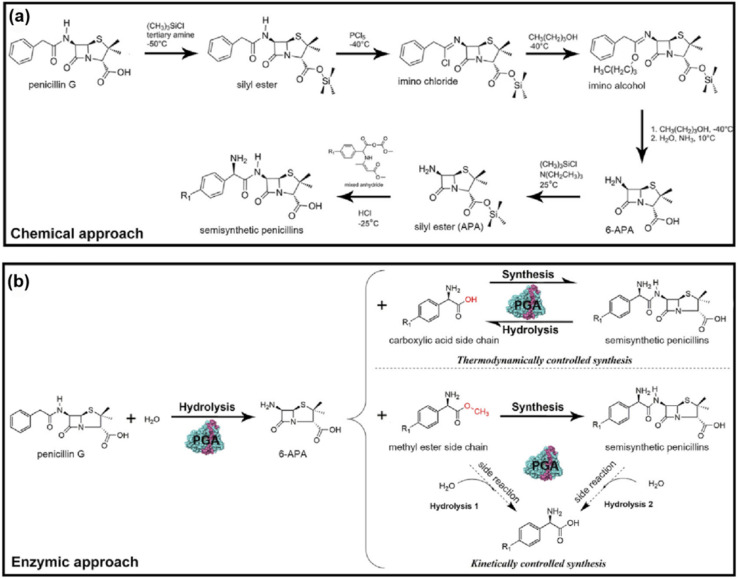
Preparation pathways for semi-synthetic penicillins: (a) traditional chemical hydrolysis of penicillin; (b) PGA-catalyzed enzymatic synthesis of semi-synthetic penicillins [Reprinted from ref. 6, which is an open-access publication licensed under CC-BY 4.0].

Conventional synthesis of semi-synthetic penicillins relies on chemical pyrolysis, as illustrated in Fig. 1a. This method requires toxic reagents like pyridine and PCl5, is performed at extremely low temperatures (−50 °C), and involves complex steps of selective functional group protection and deprotection. In contrast, enzymatic catalysis using PGA provides a more sustainable alternative (Fig. 1b). PGA catalyzes the one-step deacylation of penicillin G to produce 6-APA, a key intermediate in β-lactam antibiotic synthesis. Subsequently, PGA catalyzes the condensation of 6-APA with various acyl donors to synthesize semi-synthetic penicillins under thermodynamic or kinetic control.^7,8^ Compared to traditional chemical synthesis, this enzymatic approach offers several advantages, including milder reaction conditions, high selectivity, cost-effectiveness, and environmental compatibility. Since 1995, immobilized PGA has been widely used as an industrial biocatalyst for the large-scale hydrolysis of penicillin G to produce 6-APA, a pivotal antibiotic core.^9,10^

Furthermore, PGA has also been utilized to deacetylate cephalosporin G, a compound derived from the ring-expansion reaction of penicillin G, to produce another key intermediate, 7-amino-3-deacetoxycephalosporanic acid (7-ADCA).^7^ Subsequently, PGA catalyzes the condensation of these core structures (6-APA or 7-ADCA) with acyl donors to synthesize various semi-synthetic penicillins and cephalosporins (Fig. 1b).^4,11^

However, native (free) PGA has several limitations, including poor thermal stability, sensitivity to organic solvents, and intolerance to acidic/alkaline conditions, which severely restrict its industrial application.^12,13^ Additionally, free PGA is difficult to separate from reaction products, which limits its reusability and complicates the purification of 6-APA.^13^ To overcome these issues, researchers have adopted immobilization strategies, using carrier materials to improve PGA's environmental resilience and recyclability.^14,15^ Due to technological constraints, immobilized PGA was not successfully applied for the hydrolysis of penicillin G to produce 6-APA until the 1970s.^16^

**Fig. 2:**
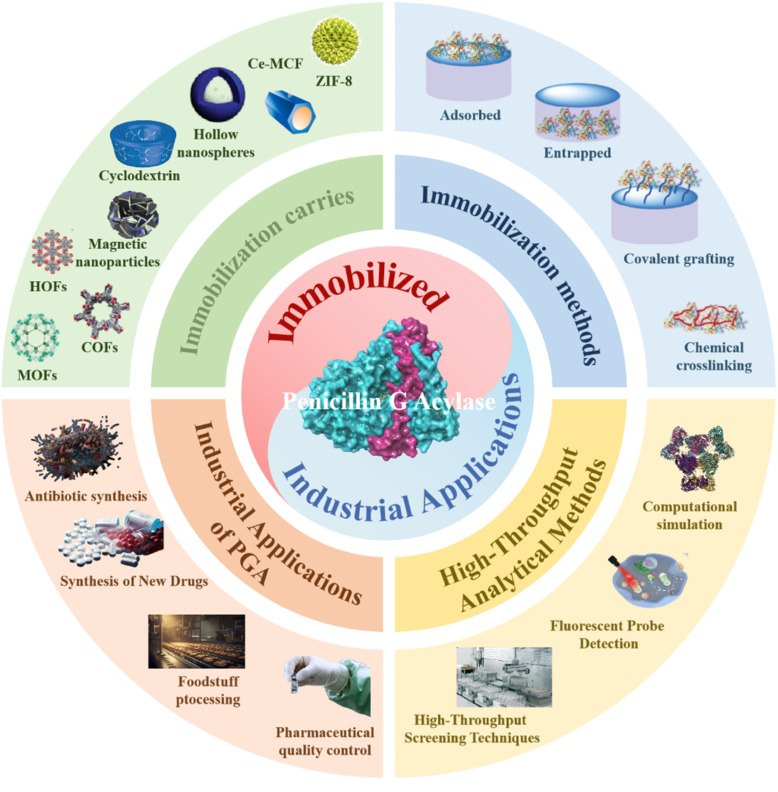
Summary of the main research framework of this paper.

Immobilization technology has been widely employed to overcome the limitations of free PGA, particularly its poor stability and lack of reusability. By anchoring enzymes onto solid supports, immobilization can significantly enhance structural stability, resistance to harsh conditions, and operational lifetime. These advantages make immobilized PGA highly suitable for industrial applications. Key performance metrics for producing 6-APA using immobilized PGA include enzyme activity recovery, loading capacity, mass transfer rate, recyclability, and operational stability. Notably, while immobilization enhances stability and reusability, it is generally accompanied by a reduction in intrinsic catalytic efficiency (kcat Km^−1^), primarily due to diffusion resistance and structural constraints. Although immobilization strengthens PGA's robustness by stabilizing its three-dimensional conformation (*e.g.*, *via* hydrogen bonding or covalent attachment), it also introduces kinetic limitations such as substrate diffusion resistance and steric hindrance. Although improved catalytic performance is sometimes reported, such enhancement generally reflects apparent activity rather than intrinsic catalytic efficiency. From a thermodynamic perspective, immobilization enhances conformational stability but may restrict the flexibility required for optimal catalytic turnover. Therefore, the overall catalytic performance reflects a balance between kinetic constraints and thermodynamic stabilization. To maximize catalytic performance, parameters such as carrier-enzyme interactions, carrier structure, microenvironment, solvent selection, and reactor design must be optimized, as these factors significantly influence PGA's conformational integrity. Recent advancements in immobilization methodologies, carrier materials, and reactor engineering have driven the development of more effective immobilized PGA systems. This review systematically examines the intrinsic properties of PGA (sources, classification, and structure) and provides a comparative analysis of current immobilization strategies, including their advantages and limitations. It also evaluates the impact of reaction media and reactor configurations on catalytic performance and concludes with an assessment of challenges and future directions in immobilized PGA technology (Fig. 2).

## 2 Penicillin G acylase

PGA, a hydrolase derived from microorganisms, is widely used in biomedical applications and serves as a vital industrial biocatalyst.^17–19^ In practical applications, PGA primarily catalyzes the enzymatic hydrolysis and synthesis of various penicillins.^17^ Through penicillin hydrolysis, PGA generates 6-APA, a key intermediate for synthesizing β-lactam antibiotics (BAs) known for their potent bactericidal activity, broad therapeutic spectrum, low toxicity, and superior clinical efficacy.^20–22^ Furthermore, PGA-mediated 6-APA production offers significant advantages over chemical methods, including enhanced safety, operational efficiency, and cost-effectiveness. Consequently, PGA has garnered substantial attention in both industrial and research domains. This section comprehensively discusses its sources, classification, structural features, catalytic mechanisms, and industrial applications.

### 2.1 Sources and classification of PGA

In the 1950s, PGA was first discovered in the mycelium of Penicillium chrysogenum Q176 by Japanese scientists Murao and Sakaguchi.^23–26^ This enzyme catalyzes the hydrolysis of penicillin G (PG) to yield phenylacetic acid (PAA) and 6-APA, with the latter existing as hygroscopic needle-like crystals exhibiting a melting point of 158–159 °C.^25,27^ Subsequently, Escherichia coli-derived PGA emerged as a key biocatalyst for the industrial production of β-lactam antibiotics.^28^ Its industrial importance largely stems from its ability to catalyze the production of 6-APA, a key intermediate for semisynthetic β-lactam antibiotics.^18,28^ Subsequent studies revealed that PGA is widely distributed across bacteria, yeast, fungi, and actinomycetes, with diverse microbial strains capable of PGA synthesis, including *Escherichia coli*, *Pseudomonas melanogena*, *Bacillus megaterium*, *Streptomyces lavendulae*, *Achromobacter*, *Gloeocercospora sorghi*, *Penicillium chrysogenum, actinomycetes*, *Proteus rettgeri*, and *Mucor griseocyanus*.^29^ Both natural wild-type and recombinant variants of these strains exhibit PGA activity, but the most efficient PGA enzymes are derived from *Escherichia coli* and *Bacillus megaterium*.

Notably, PGA produced by *Bacillus megaterium* is an extracellular enzyme, offering advantages in separation and purification compared to the intracellular PGA from *Escherichia coli*.^30^ Of particular interest, PGA from *Escherichia coli* (E.C. 3.5.1.11), with a molecular weight of 67 000 ± 1,000, (ref. 22) demonstrates broad substrate promiscuity as a non-specific acylase. It preferentially hydrolyzes PG, underscoring its significant industrial potential.

### 2.2 Structure and catalytic mechanism of PGA

**Fig. 3:**
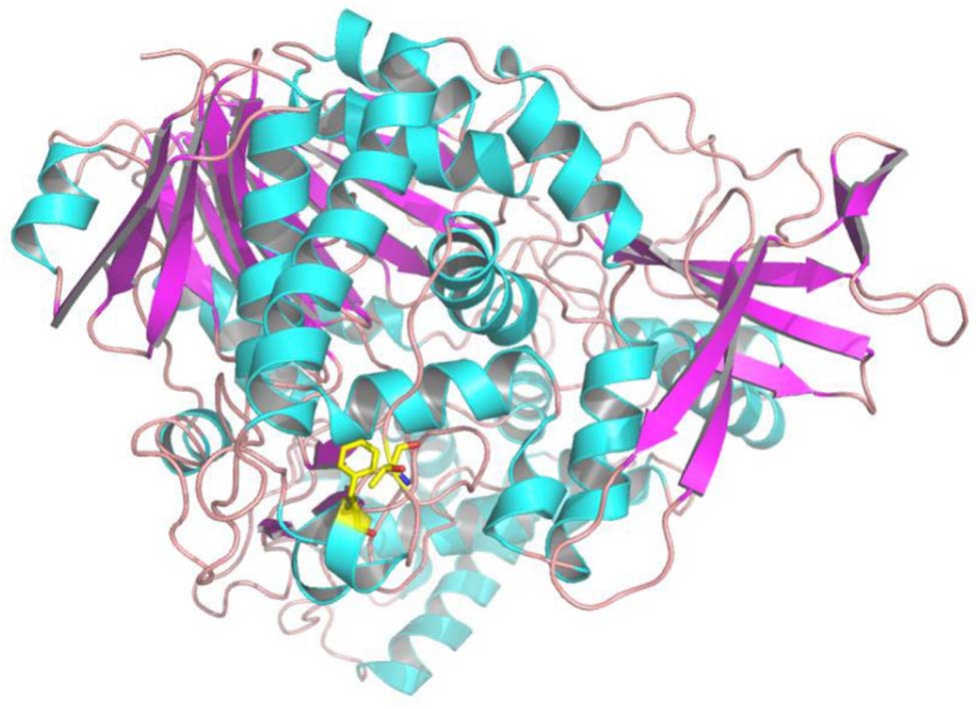
Schematic structure of PGA [Adapted with permission from ref. 33 Copyright 2003 Biotechnology Editing Department].

From a structural and mechanistic perspective, PGA belongs to the *N*-terminal nucleophilic hydrolase (Ntn) family, characterized by a canonical αββα-fold architecture. This structure comprises a four-layered catalytic αββα-core and an *N*-terminal nucleophilic residue.^31^ The core is formed by two antiparallel β-sheets stacked against each other, flanked on one side by a layer of α-helices,^32^ while the opposing face forms a hydrophobic pocket (Fig. 3 illustrates the structural schematic of PGA).^33^ The enzyme exists as a heterodimer composed of α- and β-subunits, with molecular weights of 20–24 kDa and 60–65 kDa, respectively.^19,34^

**Fig. 4:**
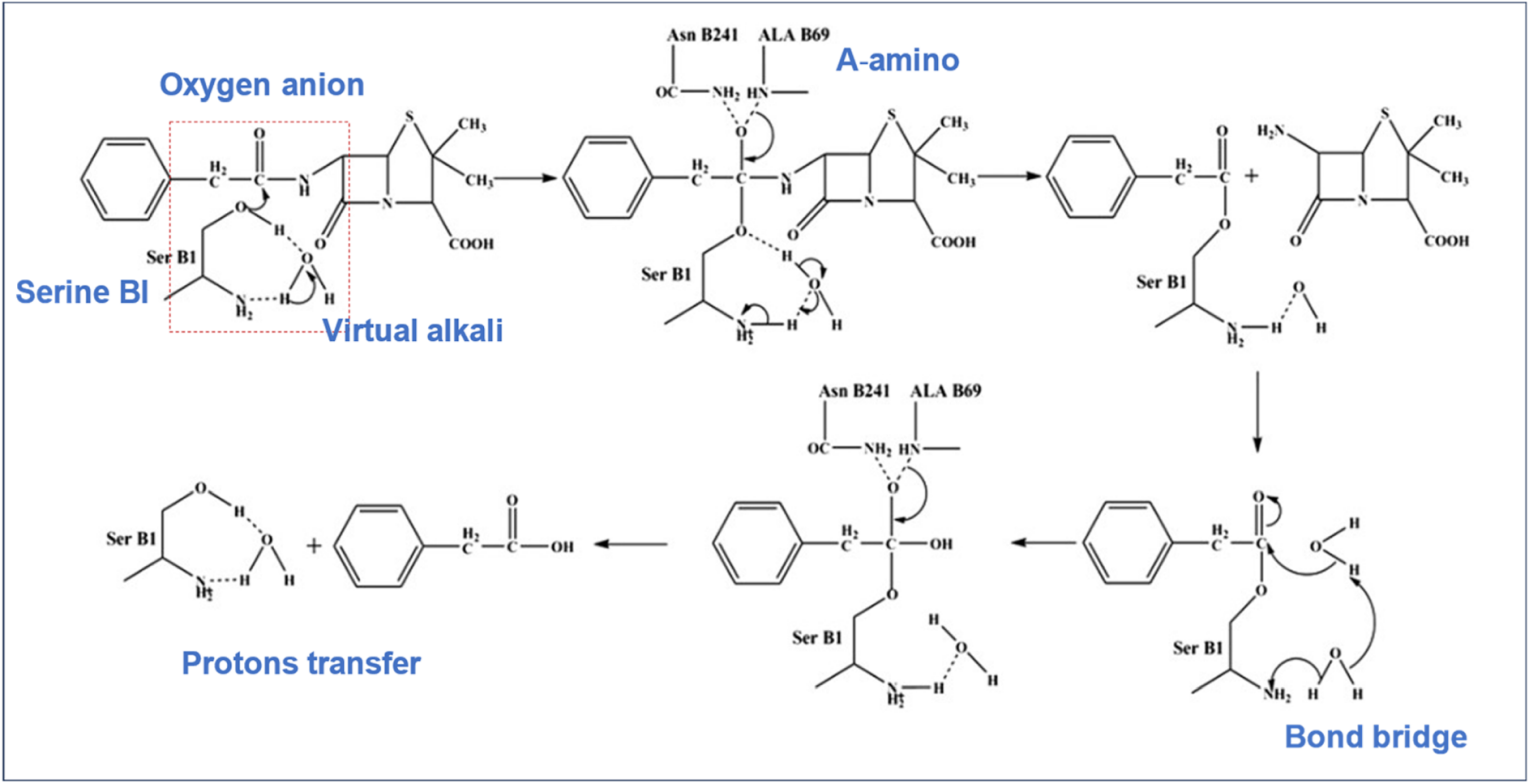
Mechanism of penicillin G potassium (PG) hydrolysis catalyzed by PGA [Adapted with permission from ref. 36 Copyright 1995 Springer Nature, License number: 6243471363994].

The catalytic mechanism of PGA involves a nucleophilic attack by the *N*-terminal serine residue (SerB1) of the β-subunit on the carbonyl carbon of the amide bond in the substrate (Fig. 4 depicts the hydrolysis mechanism of PG by PGA). This serine residue constitutes the active site, while the α-amino group of the *N*-terminal residue (located on the α-subunit) acts as a base to deprotonate the nucleophile.^19,21,35^ These substrate-specificity-determining residues are positioned within the α-subunit.

During hydrolysis, the SerB1 residue plays a critical role in cleaving the amide bond. The reaction proceeds *via* nucleophilic substitution at the carbonyl carbon of PG, forming a reversible tetrahedral intermediate stabilized by protonation of the active site carboxyl group. Notably, the absence of histidine or free carboxyl groups near SerB1 necessitates a water-mediated hydrogen-bonding network to enhance the nucleophilicity of the serine residue. In PGA's active site, the α-amino group of the *N*-terminal residue interacts indirectly with the serine hydroxyl group through a water molecule, which acts as a proton relay. This water molecule bridges the serine hydroxyl and the α-amino nitrogen, facilitating proton transfer and stabilizing the transition state. The hydrogen-bond network within the active site enables precise spatial alignment: SerB1 forms three hydrogen bonds with the tetrahedral intermediate, while the A-amino nitrogen participates in an additional hydrogen bond. These interactions direct the lone electron pair of the deprotonated serine oxygen toward the substrate, thereby accelerating nucleophilic attack. The isoelectric point of the free α-amino group (typically 6.8–7.9) ensures minimal charge interference, allowing efficient proton transfer *via* the water bridge.^37^

### 2.3 Production and culture optimization of PGA

In terms of PGA production in wild-type strains, in 2021, Cano Cabrera *et al.* utilized *Trichoderma reesei* (*Mucorgriseocyanus*) for exogenous PGA production, and showed that PGA produced by this strain was in known sequence with a high degree of identity.^38^ In addition, Borcinova in 2020 studied the production and partitioning of PGA in *Pichia pastoris*, revealing a high degree of sequence identity in the production of PGA in *Pichia pastoris*.^39^ In addition, Borcinova studied the dynamics of PGA production and secretion in *Pichia pastoris* in 2020, revealing the secretion and retention of PGA during the production process, which provided an important reference for further optimizing the production conditions.^40^ These studies showed that the performance of different wild-type strains in the production of PGA has their own characteristics, and the yield and quality of PGA can be significantly improved by selecting suitable strains and optimizing the production process.

In order to further improve the efficiency and stability of PGA, researchers have explored a lot in optimizing the production conditions. For example, PGA production has been enhanced by modulation of medium composition and addition of inducers.^41,42^ In the study of production of PGA, different culture conditions have a significant effect on enzyme activity and yield. In 2014, Elnashar *et al.* investigated the effect of three key factors on PGA immobilization by Plackett–Burman screening design, and found that enzyme buffer pH, immersion time, and concentration were the significant influencing factors.^43^ In addition, Lopes optimized the medium composition of Bacillusmegaterium in 2023, which showed a 6-fold increase in PGA activity to 135–140 IU per ml.^41,42^ Zhu catalyzed the synthesis of cefuroxime using PGA immobilized in an aqueous two-phase system (ATPS) recoverable in a study conducted in 2014, and under optimal conditions achieved 75.81% yield.^44^ These studies showed that the productivity and stability of PGA can be significantly improved by optimizing the culture conditions. In terms of its immobilization matrix, calcium alginate is a relatively affordable and biocompatible matrix for the enzymes pectinase, protease, and α-amylase among the several immobilization techniques. According to earlier studies, maltase from *Bacillus licheniform* KIBGE-IB4 was immobilized using an entrapment process within calcium alginate beads,^45^ whole cells of *Methylomonas* sp. Strain GYJ3 are now being immobilized utilizing the sol–gel techniques and sodium silicate silica precursor.^46^

The influence of entrapment in several sol–gel frameworks on enzyme stability and activity was examined using methyltrimethoxysilane as a precursor. This approach has some drawbacks, such as the enzyme periodically leaking into the solution, which reduces stability and activity. Therefore, it is crucial to modify the matrix or membrane properly. Recently, the commercial use of products processed by biocatalysts has been suggested for magnetic nanoparticles that immobilize enzymes. To reduce startup and operational costs, this approach focuses on the solid-phase magnetic characteristic, which can be swiftly retrieved from the reaction liquid and isolated using an external magnetic field.^47^ Entrapment by nanostructured substrates such as natural materials and electrospun nanofibers has revolutionized the field of enzyme immobilization, with a wide variety of applications in biofuels, fine chemistry, biosensors, and healthcare. Another study revealed that *C. rugosa* lipase entrapped in chitosan prevented friability, leaching, increased entrapment efficiency, and enzyme activity. Due to its hydrophilic nature, it is extremely affinitive to protein and non-toxic, biocompatible, and chemically modifiable.^48^

In the production and immobilization of PGA, multi-step engineering improvement is an important means to enhance its performance. In 2023, Wichmann *et al.* performed a systematic improvement of FJAT-PGA through protein engineering technology, which significantly enhanced its crystallization efficiency.^49^ This study achieved the optimization of the PGA production process by reducing the purification steps and increasing the yield, which provided strong support for further applications.

### 2.4 Industrial applications of PGA

PGA exhibits diverse industrial applications, extending beyond β-lactam antibiotic (BA) production to include roles as a biocatalytic component in peptide synthesis and the resolution of racemic mixtures in chiral compound preparation,^33,50^ the detailed application summary is shown in Table 1. In antibiotic manufacturing, BAs account for over 50% of clinical antibiotic usage due to their high efficacy and broad-spectrum activity. Most BAs are derived from 6-APA, a semi-synthetic intermediate traditionally produced *via* chemical methods. However, conventional chemical synthesis of 6-APA suffers from critical limitations: (a) complex reaction protocols requiring sequential protection and deprotection of functional groups. (b) Hazardous reagents such as pyridine, PCl_5_, dimethylaniline, dichloromethane, and NOCl. (c) Non-degradable waste generation. (d) High energy consumption. In contrast, PGA-catalyzed enzymatic hydrolysis offers a sustainable and efficient alternative for 6-APA production. As early as 1990, (ref. 51) industrial-scale 6-APA synthesis *via* PGA-mediated PG hydrolysis was successfully implemented, establishing enzymatic methods as the dominant route for this critical intermediate.

**Table 1:** Summary of different applications of immobilized enzymes

Enzyme	Applications
Laccase from *Pyricularia oryzae*	Fungicide for controlling rice smut disease
*Candida molischiana* glucomylase	Detect sugar and glycoside substances, and produce oligosaccharides
β-Galactosidase	Hydrolyzed dairy products, whey and galactooligosaccharides
Pectinase from *Aspergillus aculeatus*	Promote seed growth, prepare plant protoplasmic materials
Pectin lyase	Promote seed growth, prepare plant protoplasmic materials
β-Galactosidase and amyloglucosidase	Hydrolyze lactose in whey/whey permeates, skimmed milk
Lipase from *Candida rugosa*	Production of oil and grease
Tyrosinase	Detect phenolics in red wine
Cardosin a (protease)	α-Lactoglobulin production
Pectinase	Pectin solution production
β-Galactosidase	Removal of lactose from milk
Trypsin	β-Lactoglobulin production

The selectivity and stereospecificity of PGA make it the catalyst of choice for obtaining products.^52^ However, recycling and isolating from the substrate and products in the reaction medium is difficult, increasing operational difficulties and economic expense but also causing contamination of the products and the environment, which naturally impacts industrialization. Furthermore, free PGA has poor environmental tolerance due to its exceedingly unstable conformation.^22^ During operation, it is sensitive to temperature and pH, which eventually causes a considerable decline or even loss of enzyme production. These elements increase operational costs and complexity in industrial applications.^53^ Enzyme immobilization technology was developed in the 1960s to address the downsides of free enzymes. Compared to soluble PGA, immobilized PGA has significantly improved operational stability, increased enantiomeric selectivity, reusable enzyme, simple reactor operation, and to some extent, easy product separation.^54–56^ The immobilized PGA technology is described in detail below (Table 2).

**Table 2:** Immobilized PGA with significant industrial implications

Enzyme source	Support	Reference
*E coli* PGA	Polymer-coated glyoxyl-agarose	Hilbert, 2021
*E. coli* PGA	Grafted nylon membranes	Zuza *et al.*, 2007
*E. coli* PGA	Mesoporous silica	Karatus *et al.*, 2021
*E. coli* PGA	Acrylamide copolymer	Hassan 2016
*E. coli* PGA	Cross-linked aggregates	Ye *et al.*, 2019
*E. coli* PGA	Sepabeads	Zuza *et al.* 2007
*E. coli* PGA	Activated chitosan	Chen *et al.*, 2022
*E. coli* PGA	Sol–gel silica xerogel	Bernardino *et al.*, 2011
*E. coli* PGA	Heterofunctional disulfide epoxide	Grazu *et al.*, 2012
*Acetobacter turbidans* PGA	Eupergit C	Pitkina., 2015
*Providencia rettgeri*	Methacrylic polymers	Ali *et al.*, 2020
*Bacillus badius* PGA	Cross-linked aggregates	Grulich *et al.*, 2013
aOU	Cross-linked acrylate	Karnes *et al.*, 2020
OU	Cross-linked carragenen	Liew *et al.*, 2019
OU	Superparamagnetic silica microspheres	Lee *et al.*, 2010
OU	Amine-activated polyvinyl chloride	Madina-castillo *et al.*, 2022

a PGA, penicillin G acylase; *OU (origin unknown) indicates that the source of the enzyme was not mentioned in the referenced publication.

#### 2.4.1 Applications in antibiotic synthesis

In terms of applications in antibiotic synthesis, PGA plays a crucial role as a key enzyme in the production of β-lactam antibiotics. In 2014, Maresova noted that PGA is of significant importance in industrial applications and that its annual consumption is very high.^57^ Cobos Puc's 2020 study further emphasizes the critical role of PGA in the production of widely used beta-lactam antibiotics.^58^

In recent years, advances in biotechnology have greatly enhanced the potential for industrial applications of PGA. Srirangan's 2013 study examined the importance of PGA in the production of semi-synthetic antibiotics and noted that advances in biotechnology have enhanced its use in industry.^59^ Pan's research in 2022, on the other hand, suggests new strategies that face challenges but demonstrate their great potential for industry.^6^ In addition, important advances have been made in the production and characterization of PGA for specific strains. For example, showed in 2025 that *Bacillus thuringiensis* BGSC BD1 was the best PGA producer, with its activity remaining stable at pH 7–8 and up to 60 °C.^60^ Deng's modification of *Alcaligenes faecalis* PGA by saturation mutagenesis in 2015 significantly improved the production of ampicillin, increasing productivity by more than 130-fold. (ref. 61) In addition, the development of new strains and mutants opens up new possibilities for the application of PGA. In another study by Pan in 2020, efficient synthesis of β-lactam antibiotics was achieved using the newly isolated *Achromobacter xylosoxidans* PX02 PGA mutant, simplifying the purification process.^62^ In summary, the application of PGA in antibiotic synthesis not only relies on its own efficient catalytic ability, but also benefits from the advances in biotechnology, the optimization of specific strains and the development of immobilization technology. These studies have laid a solid foundation for the future wide application of PGA in industrial production.

**Fig. 5:**
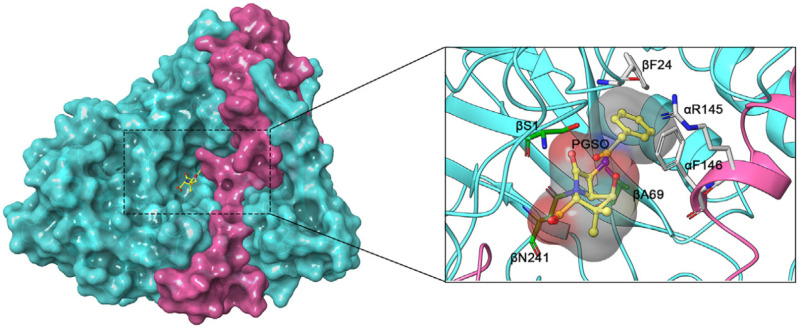
EcPGA crystal structure (PDB ID: 1GM9) of ligand penicillin G sulfoxide (PGSO) (yellow). α and β subunits are purplish red and cyan, respectively. The active sites βS1, βA69 and βN241 are green, and the good mutant sites αR145, αF146 and βF24 are light gray [Reprinted from ref. 64 which is an open-access publication licensed under CC-BY 4.0].

Protein engineering is used as an effective and feasible strategy to improve the efficiency of enzymatic antibiotic synthesis by improving the intrinsic properties of PGAs. In the case of EcPGA, its active sites are composed of amino acid residues βS1, βA69, and βN241 (ref. 36 and 63). According to the mutation assessment, some residues close to the binding site, such as αR145, αF146, and βF24, have strong interactions with the substrate and are therefore considered good mutation targets^64^ (Fig. 5). According to the enzyme-induced fitting model, EcPGA binds to large substrates (*e.g.*, penicillin G) with a structural transition from a closed conformation to an open conformation, which is mainly driven by the fact that αR145 and αF146 are far away from the binding site. Specifically, αF146 can help the thiazolidine ring of the substrate to bind *via* van der Waals interaction, whereas αR145 binds to the carboxylic acid oxygen atom of the substrate *via* two or three water molecules.^64^ βF24 residue also plays an important role in the substrate binding because of the presence of this aromatic residue with the benzene ring of the substrate hydrophobic interaction.^65^ Therefore, most studies on pages targeted mutagenesis have been based on αR145, αF146 and βF24.

#### 2.4.2 Pharmaceutical quality control

The development of new drugs has been significantly advanced by research on immobilized PGA. This biocatalyst has proven to be instrumental in two key areas: the synthesis of novel antibiotics and analytical applications in drug detection.

In the field of drug synthesis, immobilized PGA serves a critical role in the production of semi-synthetic penicillins,^66^ offering substantial economic benefits through simplified preparation methods and expanded applications.^37^ For example, Knezevich effectively improved PGA's performance by immobilizing it on a macroporous poly(GMA-*co*-EGDMA) carrier, enhancing its use in drug processing.^67^ Additionally, a monomer reactor utilizing a polyaniline (PANI) coated capillary column was developed by Ahn for the synthesis of cefhydroamphenicol, demonstrating a rapid reaction rate and excellent operational stability.^68^

Beyond synthesis, immobilized PGA shows great promise as a rapid and sensitive tool for chiral separation and drug detection. Gotti *et al.*, for instance, utilized an epoxy silica gel-integrated capillary column as an immobilized carrier to achieve efficient separation and quantitative analysis of aromatic acid-type chiral drugs. This method not only enabled the rapid separation of compounds like ketoprofen, flurbiprofen, and sulprofen but also provided a highly sensitive means of quantitatively analyzing the active enantiomer (S)-ketoprofen in commercial tablets.^69^ These findings collectively underscore the significant potential of immobilized PGA in both antibiotic manufacturing and drug quality control.

## 3 Immobilization technique of PGA

Enzyme immobilization has been widely recognized as an effective strategy to enhance enzyme stability under harsh operational conditions.^55^ Generally,^56^ immobilizing PGA through multiple binding sites (*e.g.*, subunits, –NH_2_ functional groups) on a carrier prevents subunit dissociation, protease aggregation, autolysis, or proteolysis, thereby stabilizing its structural integrity. The catalytic performance of immobilized PGA is governed by the interplay between kinetic and thermodynamic factors. From a kinetic perspective, immobilization may introduce mass transfer resistance and diffusion limitations, which restrict substrate accessibility to active sites and reduce observed reaction rates. From a thermodynamic perspective, immobilization enhances enzyme stability by stabilizing its conformation and creating a favorable microenvironment, such as regulated local pH and polarity. Therefore, the overall performance of immobilized PGA reflects a balance between kinetic limitations and thermodynamic stabilization. Although improved catalytic performance is sometimes reported, such enhancement generally reflects observed activity rather than intrinsic catalytic efficiency (kcat Km^−1^).

This leads to improved operational stability, reusability, and cost-effectiveness. Furthermore, immobilization can modulate the geometric configuration of PGA's active site, which may enhance substrate selectivity and stability, while catalytic activity is typically maintained or reduced depending on immobilization conditions. It also improves conformational stability while protecting the enzyme from degradation by organic solvents, elevated temperatures, or other adverse environmental factors.^70^ Surface functionalization and cross-linking techniques can further improve enzyme stability and maintain catalytic activity.^67,71^ Immobilization inherently involves a trade-off between catalytic efficiency and operational stability. While structural stabilization improves enzyme robustness, kinetic limitations such as mass transfer resistance and steric hindrance may reduce intrinsic catalytic efficiency. Therefore, the design of immobilized systems should aim to minimize kinetic constraints while maximizing structural stabilization. In addition, high-throughput screening and computational modeling-based approaches have been used to analyze and improve the catalytic performance of PGAs.^72^ These advantages are closely related to the immobilization strategies and carrier materials employed. Recent advancements in PGA immobilization techniques and carrier design have been extensive, as discussed below.

### 3.1 Immobilization methods

**Fig. 6:**
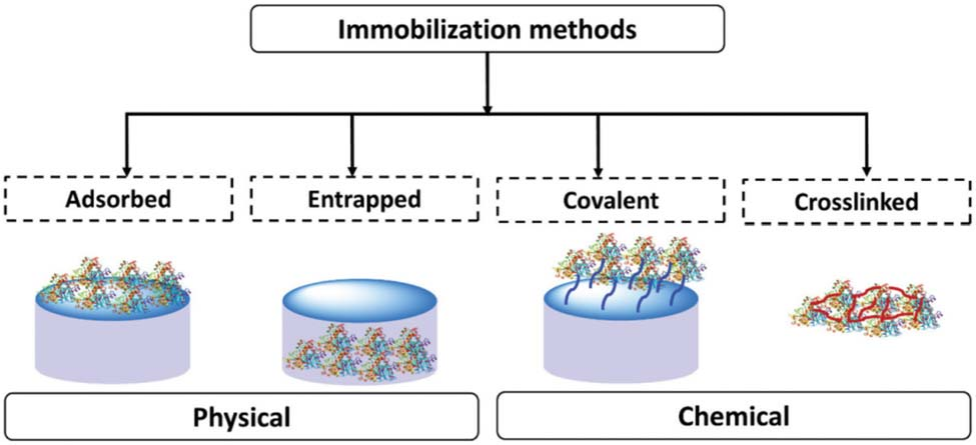
Classification of enzyme immobilization methods [Adapted with permission from ref. 76 Copyright 2018 Springer Nature. License number: 6243480946213].

A vast segment of immobilization techniques can be categorized using various methods, frequently divided into reversible and irreversible categories. The ease of reversal is inversely correlated with the strength of the enzyme's binding to the adsorbent. It can also be categorized based on the chemical or physical process used to immobilize it. Reversible or physical methods or: (a) disulfide bond formation and (b) adsorption. Irreversible or chemical modification methods: (a) entrapment, (b) cross-linkage, and (c) covalent bonding. Because vectors and enzymes have various properties, it is challenging to develop a universal immobilized enzyme manufacturing process.^73^ The immobilization method in the process of immobilizing PGA is an important issue that must be considered, which is simply a linkage between PGA and vector,^73^ making PGA insoluble and recoverable. Until recently, the combination of PGA and functional groups on the carrier has been broadly classified as physical and chemical. The physical method refers to the immobilization of PGA by physical interaction with the carrier material (without forming any chemical bond). PGA can be either adsorbed on the surface of the carrier material or placed inside the carrier material. Chemical methods mostly involve PGA being cross-linked or covalently bonded to the support material (Table 2). The techniques can, of course, be further subdivided based on the properties they share. Physical techniques, such as adsorption and embedding,^74^ are reversible immobilization; chemical techniques, such as covalent and cross-linking,^75^ are irreversible. Immobilization methods are classified as shown in Fig. 6 (ref. 76). This section provides a brief description of immobilization methods.

#### 3.1.1 Adsorption method

Among the many protein immobilization methods, the most common and simplest is adsorption immobilization,^77^ whose advantage also lies in its versatility: the method can be used for different types of enzymes, catalyzing various reactions,^78^ and of course, PGA, when certain conditions are met. Among other driving forces, the approach relies heavily on physical connections between the carrier and the enzyme, such as van der Waals contacts, ionic interactions, hydrophobic interactions, hydrogen bonds, and electrostatic interactions, which preserve enzyme structure (thermodynamic effect) but may still be influenced by diffusion limitations (kinetic effect). It is important to note that such binding is typically weak and does not alter the intrinsic structure of the enzyme, preventing disruption of the enzyme's active site. This assists in maintaining the enzyme's catalytic properties and makes it easier to transport the substrate to its active center.^79^ Through weak interactions, many researchers have immobilized enzymes on carriers such as polysaccharide derivatives, synthetic polymer resins, activated carbon, silica, glass and ceramics. Among them, mesoporous silica materials are of particular interest.

**Fig. 7:**
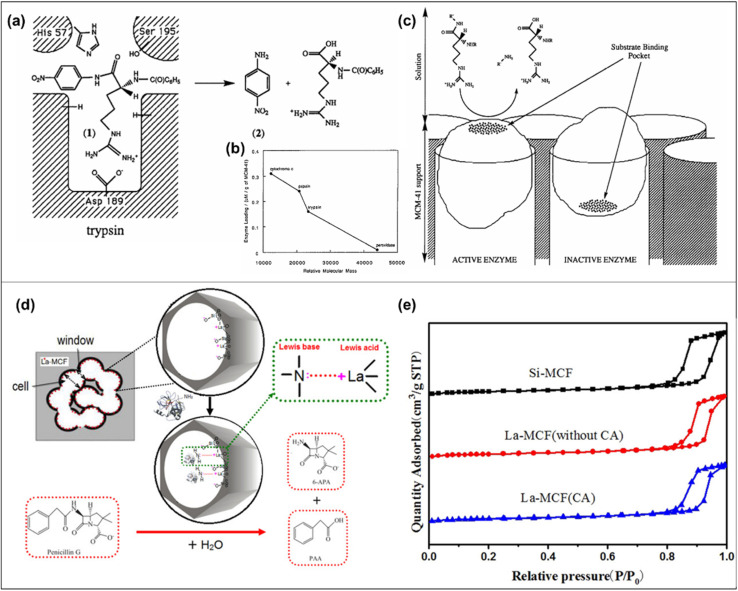
Mesoporous silica materials as immobilization carrier. (a) and (c) Schematic diagram of the effect MCM-41 immobilized trypsin. (b) Effect of enzyme size on the immobilization the MCM-41 host [Reprinted with permission from ref. 70. Copyright 1996 Elsevier. License number: 6243481464187]. (d) Proposed mechanism for the immobilization of PGA on La-MCF (with CA) and hydrolysis of penicillin G potassium salt catalyzed by PGA. (e) Nitrogen adsorption–desorption isotherms of Si-MCF, La-MCF (without CA) and La-MCF (with CA) [Reprinted with permission from ref. 82 Copyright 2022 Taylor & Francis].

According to Diaz *et al.*,^70^ there is a noticeable enzyme size dependence in the physical adsorption of pure silica MCM-41 (ref. 80) with hexagonal phase (Fig. 7a–c). Xue *et al.*^80^ immobilized PGA on MCM-41, MCM-48, and Co-MCM-48 molecular sieves and found that Co-doped carriers exhibited superior stability. After storage, Co-MCM-48 (Co/Si = 0.01 and 0.02) retained 84.5% and 81.7% of their initial activity, respectively, significantly higher than those of MCM-41 and MCM-48. Tian *et al.*^81^ found that incorporating lanthanum into mesoporous silica foam enhances enzyme immobilization and increases the activity of PGA due to the interaction between Lewis acids and bases. The enzyme was immobilized through strong interactions between the Lewis acid sites on the La-MCF support and the free lysine amino groups of PGA (Fig. 7d and e). The immobilized PGA exhibits high specific activity and enhanced operational stability. This method offers the potential for developing innovative immobilized enzyme catalytic systems with high stability. Tu *et al.*^82^ synthesized Fe_3_O_4_@PTA-GA NPs and Fe_3_O_4_@PTA NPs for the physical immobilization of PGA, the results revealed that the latter was excellent. Compared to the Fe_3_O_4_@PTA NPs with physically immobilized PGA, the Fe_3_O_4_@PTA-GA NPs demonstrated greater catalytic stability, reusability, and storage stability.

#### 3.1.2 Encapsulation method

Other techniques for physically immobilizing enzymes, mainly by encapsulating them in polymer networks/gel lattices (*e.g.*, organic polymers, silica-sol-gels) and membrane devices (*e.g.*, hollow fibers, microcapsules).^83^ Unlike adsorption methods, this method does not locate the enzyme directly on the surface of the carrier. Instead uses physical methods to confine the enzyme or encapsulate it in a polymeric matrix.^84^ The key advantage of the carrier is that it does not require excessive interaction with the protein. Moreover, to some extent avoids the negative impact of the rigidity of the material on the fragile enzyme structure and protects it from harsh environments,^76,85^ enhancing thermodynamic stability, although diffusion through the matrix may introduce kinetic limitations, making it more stable and easier to isolate and recover.^77,86^ Due to this, the encapsulated PGAs often exhibit good enzymatic activity, thermal stability and pH stability.^87^

**Fig. 8:**
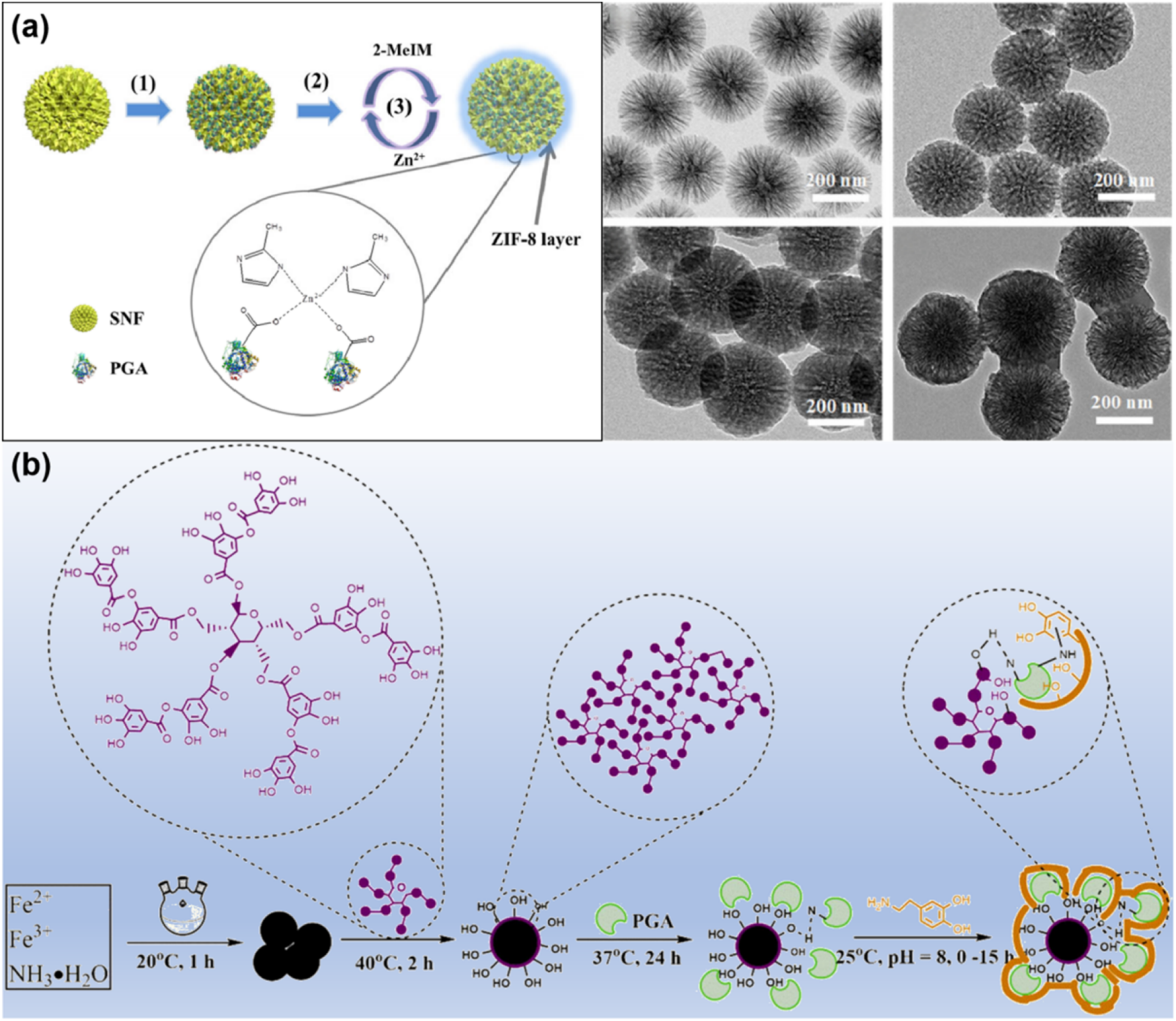
(a) Preparation process and the micro structure of PGA@SNF@ZIF-8 [Adapted with permission from ref. 76 Copyright 2018 Springer Nature. License number: 6243480946213]. (b) Preparation process of Fe_3_O_4_@PTA-PGA/PDA sandwich-like structured nanocapsules [Reprinted with permission from ref. 88 Copyright 2022 Elsevier. License number: 6243490616588].

Du *et al.*^76^ prepared the first MOF protective layer on the surface of the immobilized enzyme by first immobilizing PGA molecules on the surface of silica nanoflower folds and controlling the bionic growth of the MOF layer on its surface to achieve encapsulated immobilization (PGA@SNF@ZIF-8). The nanobiotocatalyst system may diffuse substrates and products both inside and outside of it because of its porous MOF layer (Fig. 8a). This biocatalyst prepared by separating the MOF layer from the external stress conditions exhibited better temperature stability, maintaining 78.19% of the initial viability after 10 consecutive iterations. The enzyme activity remained at 84.66% after 28 days of storage at 4 °C. Inspired by the sandwich-like structure, Liu *et al.*^88^ first physically immobilized PGA in tannic acid (TA) substrate. The polydopamine (PDA)-coated Fe_3_O_4_@PTA-PGA/PDA nanocapsules were made by physically immobilizing PGA on tannic acid (TA) self-functionalized iron tetroxide nanoparticles and then post-modifying them with spontaneous *in situ* self-polymerization of dopamine (DA) (Fig. 8b). The findings demonstrated that Fe_3_O_4_@PTA-PGA with PDA protection significantly outperformed Fe_3_O_4_@PTA-PGA without PDA protection in terms of storage stability, thermal stability, and pH stability. In addition, Fe_3_O_4_@PTA-PGA/PDA nanocapsules maintained 94.1% of the initial enzyme activity after 12 repeated uses. However, as with physisorption, the enzyme is physically confined in the carrier material and cannot be completely prevented from leaking without forming chemical bonds due to the relatively weak interaction forces. Hence, enzyme leakage must be balanced with mass transfer limitation when utilizing this method.^89^

#### 3.1.3 Covalent method

One of the key strategies for immobilizing enzymes is covalent binding. The enzyme can be firmly immobilized on the surface of the carrier using the properties of strong and stable covalent bonds,^90^ which means that the enzyme is less likely to be stripped during successive use, reflecting enhanced thermodynamic stability, although restricted flexibility may affect catalytic efficiency.^91^ Furthermore, this approach ensures that the carrier has a high loading efficiency,^92^ making it more suitable for repeated cycling and ideal for industrialization. The method's basic idea is simple: under the right circumstances, amino acids react with an insoluble carrier with an active group to produce covalent bonds.^77^ In order to achieve PGA immobilization, the functional groups of PGA^93^ are covalently attached to the functional groups of the carrier.^22^ In order to assure the robust binding of PGA to the carrier, it is therefore always a vital study direction for scientists to further increase the activity of immobilized PGA by covalent binding approach.^93^

**Fig. 9:**
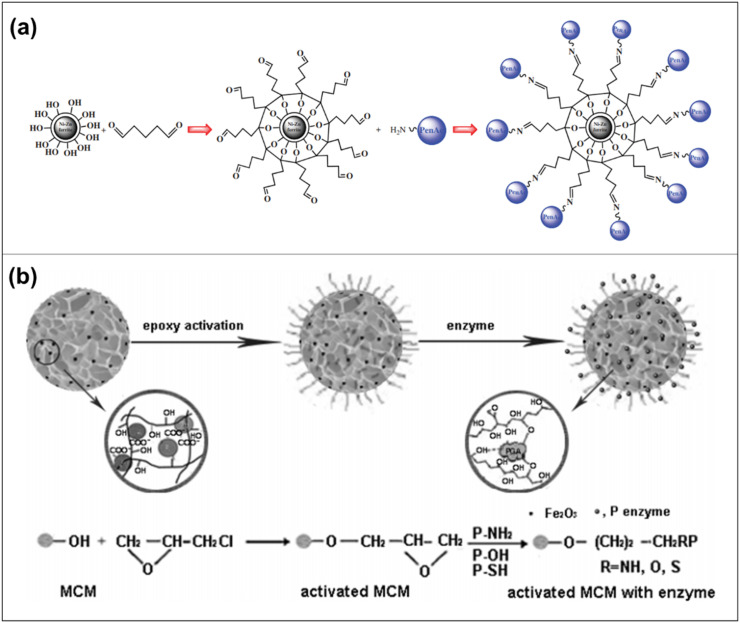
(a) Immobilization process of PGA on magnetic Ni_0.5_Zn_0.5_Fe_2_O_4_@SiO_2_ nanocomposites [Adapted with permission from ref. 95 Copyright 2016 American Scientific Publishers. Permission required]. (b) Activation of epoxy resin in magnetic cellulose microspheres and the immobilization of PGA processes [Reprinted with permission from ref. 97 Copyright 2010 American Chemical Society. License number: 6243500699282].

This covalent binding of enzymes to carrier materials involves two main steps:^94^ synthesizing or modifying the carrier material by adding reactive compounds and its further functionalization to obtain immobilized targets. Liu *et al.*^95^ successfully prepared core–shell magnetic Ni_0.5_Zn_0.5_Fe_2_O_4_@SiO_2_ nanocomposites using a facile sol–gel combustion and gel calcination process, followed by their surface functionalization by glutaraldehyde to immobilization of PGA was achieved (Fig. 9a). The outcomes demonstrated that the immobilized PGA had a high level of enzymatic activity, good catalytic stability, and the ability to sustain 63.5% of the initial activity throughout 12 applications. Similarly, Tu and Zhang *et al.*^82,96^ covalently immobilized PGA by coating tannic acid (TA) and dopamine (DA) on the surface of Fe_3_O_4_ nanoparticles, respectively, and then grafting glutaraldehyde (Fe_3_O_4_@PTA-GA NPs and Fe_3_O_4_ @PDA-GA NPs), thus achieving covalent immobilization of PGA with similarly good results. However, the Schiff base reaction using glutaraldehyde with –NH_2_ on PGA may cause irreversible deformation of the active center of the enzyme resulting in reduced enzyme activity,^76^ which deserves further attention. It has also been discovered that when the epoxide group in the carrier attaches to the –NH_2_ in a click reaction, the configuration of the PGA catalytic site is less likely to be affected. Luo *et al.*^97^ used epichlorohydrin-activated cellulose microspheres MCMs to coat spherical magnetic γ-Fe_2_O_3_ nanoparticles, which were later used for immobilization of PGA (Fig. 9b) with nearly 100% recovery of enzymatic activity.

#### 3.1.4 Cross-linking method

**Fig. 10:**
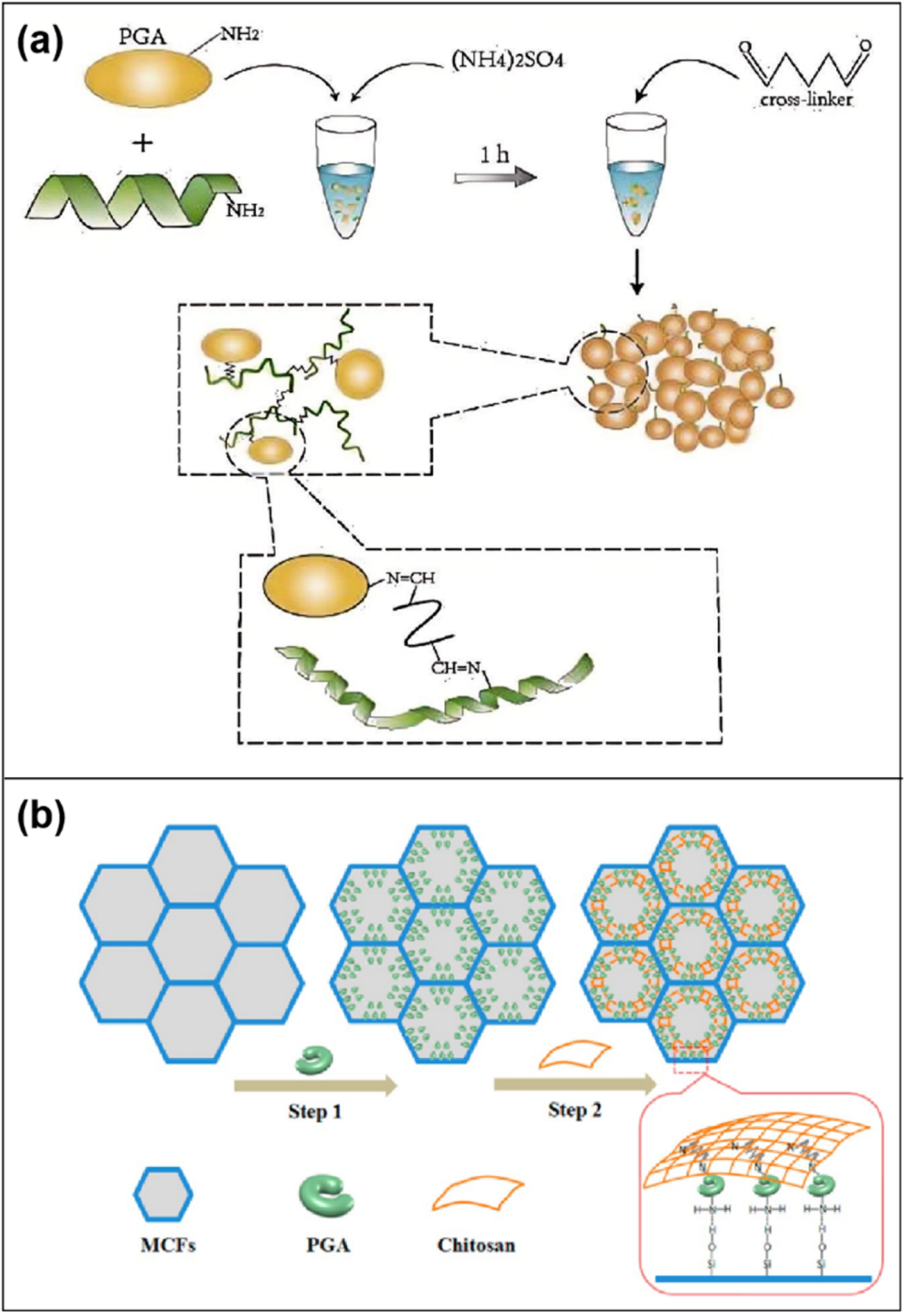
(a) Poly-lysine supported cross-linked enzyme aggregates of PGA and its application in synthesis of β-lactam antibiotics [Adapted with permission from ref. 104 Copyright 2019 Elsevier. License number: 6243500849708]. (b) Cross-linked network immobilization of PGA process [Reprinted with permission from ref. 71 Copyright 2014 American Chemical Society. License number: 6243500977850].

A carrier-free immobilization technique called cross-linking produces two immobilization products: cross-linked enzyme aggregates (CLEAs) and cross-linked enzyme crystals. This method's simple processes mostly include precipitation and cross-linking.^98^ The physical aggregation of protein molecules to form supramolecular structures can be induced by adding salts, organic solvents, or non-ionic polymers to the protein solution.^99^ This is followed by forming a double/multifunctional reagent between covalent bonds and the b/multifunctional reagents to obtain a cross-linked framework structure,^100,101^ which leads to the immobilization of the enzyme. The benefits of employing this approach to immobilize PGA are equally substantial. To begin with, immobilization without a carrier typically lowers production costs; additionally, this immobilized PGA has a higher enzymatic activity, which can successfully prevent the dilution issue of the immobilized enzymatic activity on the carrier, encourage activity recovery, and produce an increased specific activity, resulting in higher catalytic performance.^102,103^ Ye *et al.*^104^ prepared poly(lysine-loaded) PGA cross-linked enzyme aggregates (PL-CLEAs), which maintained 83% of the free enzyme activity by adding polylysine. However, due to cross-linking between PGAs, catalytic sites of PGAs are easily affected, which may introduce kinetic limitations, while the cross-linked structure improves thermodynamic stability.^105,106^ As a result, rather than being employed alone, cross-linking is frequently utilized as an adjuvant to adsorption or embedding procedures during immobilization.^106^ Shi *et al.*^71^ first used mesoporous silica foams (MCFs) to adsorb PGA physically, and then added chitosan and glutaraldehyde to the cross-linked immobilized PGA (Fig. 10), which successfully prevented the leaching of enzymes. In addition, immobilized PGA showed high operational stability in terms of pH and thermal stability.

Despite extensive progress in immobilization strategies, their practical performance varies significantly. Adsorption methods preserve enzyme structure but suffer from weak binding and enzyme leakage. Covalent immobilization enhances stability but may reduce catalytic efficiency due to restricted flexibility. Encapsulation provides a protective environment but often introduces diffusion limitations. Cross-linking improves structural rigidity but may compromise active site accessibility. Overall, no single strategy simultaneously optimizes activity, stability, and mass transfer, highlighting the need for rational design that balances kinetic and thermodynamic effects.

### 3.2 Immobilization carrier

The material used to immobilize the enzyme is called the carrier or support matrix. The properties of the enzyme and the carrier material determine the parameters of the immobilized enzyme. An enzyme's distinct chemical, metabolic, mechanical, and kinetic characteristics are produced through their interaction.^107^ As a result, the carrier's qualities are critical to the effectiveness of the immobilized enzyme. The ideal carrier includes the following properties: (a) low cost and environmentally friendly, with a reduced economic impact on the process and high renewability at the end of the immobilized enzyme's useful life; (b) completely inert after immobilization, without hindering the desired reaction; (c) with good thermal and mechanical properties, allowing the immobilized enzyme to be used under a variety of operating conditions; (d) a specific surface area that meets a certain loading capacity; (e) possess good biocompatibility and high enzyme affinity; and (f) have antibacterial and non-specific adsorption ability.^108^

As of now, loading, enzyme activity, reaction rate, and reuse are significant indicators, reflecting the combined effects of mass transfer (kinetic) and structural stability (thermodynamic). Therefore, one of the most popular study topics in this area has been building carriers with appropriate composition and structure. Currently, immobilized carriers can be classified into inorganic, organic, and composite carriers according to the composition of carriers. The following is a discussion of the progress of their research.

#### 3.2.1 Inorganic carrier

A variety of inorganic solids (such as silica, zeolites, metal oxides, zeolites, mesoporous silica, *etc.*) can be used for enzyme immobilization because of their good heat resistance, corrosion resistance, high mechanical strength, cost-effectiveness, uniform pore size distribution, and large specific surface area. Nowadays, silica materials are one of the simplest and cheapest carriers for immobilized enzymes.^66^ Among them, mesoporous silica (often called nanosilica) has been widely used for enzyme immobilization due to its very high surface area (300–1500 m^2^ g^−1^).^87^ They serve as effective PGA carriers, increasing the loading and response rate (or mass transfer rate) of immobilized PGA (Table 3). Although they have many hydroxyl groups on their surfaces, they have fewer functional group types, which somewhat restricts the immobilization approach of PGA. Researchers grafted functional groups onto inorganic materials such as SH, –CN, –NH_2_, –CHO, *etc.*, to address the abovementioned issues and then employed covalent binding with PGA functional groups to achieve immobilization.^109^

**Table 3:** Comparison of immobilization of enzymes with bioactivity

Author	Classification	Examples
Fang *et al.*, 2011	Inorganic polymers	Silica, lassy carbon, PANCMA nano-fibers, processed materials, PANCMPC
Hormigo, 2009		
Mahajan *et al.*, 2010	Organic polymers (natural polymers)	Agarose, calcium alginate, starch polymeric blend, chitosan, gelatin succinylated gelatin, protein: collagen, carbon
Hanachi *et al.*, 2015		

**Fig. 11:**
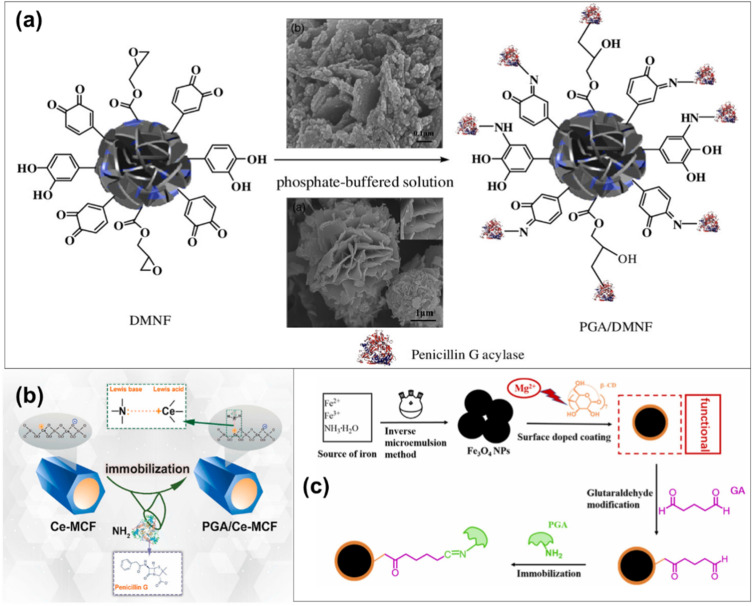
Magnetic nanoparticles for immobilized enzymes. (a) Schematic representation of the covalent interaction of PGA with DMNFs [Adapted with permission from ref. 112 Copyright 2020 Wiley-VCH GmbH. License number: 6243510059760]. (b) Cerium (iv)-Incorporated mesostructured cellular foam for immobilization of PGA [Adapted with permission from ref. 115 Copyright 2021 Elsevier. License number: 6243510210807]. (c) Preparation scheme of immobilized PGA by Mg^2+^-Fe_3_O_4_@β-CD-g-GA [Adapted with permission from ref. 116 Copyright 2023 Elsevier. License number: 6243510357624].

Sun *et al.*^110^ modified mesoporous silica samples with optimal pore size of about 11.5 nm with different amounts of epoxypropyltrimethoxysilane to fabricate surface epoxy-functionalized mesoporous silica samples with different densities of epoxide groups and finally covalently immobilized PGA with a loading amount of 112 mg g^−1^. Chong *et al.*^111^ used the nonionic triblock copolymer P123 as a template for the immobilization of PGA by co-condensation of the macroporous nanoporous material SBA-15 functionalized with organoilanes. It was shown that various organosilane densities in the range of 0.5–2.6 mmol g^−1^ showed better immobilization of penicillin acylase due to the enhanced surface hydrophobicity and electrostatic interaction of the functional groups with the enzyme. In addition, magnetic carriers have attracted much attention in immobilizing PGA in recent years because magnetic carriers can be simply and quickly separated from the mixed reaction system under magnetic force, avoiding the loss of biocatalysts during the mechanical operations of separation and rinsing. Zheng *et al.*^112^ designed novel bifunctional magnetic nano-flowers (DMNFs), which have both epoxy groups and hydrophilic catechol and phthaloquinone groups for covalent immobilization of PGA (Fig. 11a). The results showed that after 10 cycles, 106 mg g^−1^ of enzyme loading maintained more than 90% of the initial activity.

##### 3.2.1.1 Magnetic nanoparticles

Magnetic nanoparticles, as a typical inorganic material, are one of the most widely used PGA immobilization carriers. In the study of immobilization on magnetic nanoparticles, significant progress has been made in recent years. In the study of immobilization on magnetic nanoparticles, significant progress has been made in recent years. In 2016, Liu *et al.*^113^ achieved high activity, stability and ease of separation of PGA by surface-modified NiFe_2_O_4_ nanorods. Also in 2016, Ling *et al.*^114^ immobilized PGA on Fe_3_O_4_@chitosan magnetic nanoparticles, which not only improved efficiency and stability, but also successfully synthesized amoxicillin. Chen *et al.*^32^ synthesized paramagnetic polymer microspheres on the surface of silica-coated Fe_3_O_4_ nanoparticles by back-suspension polymerization using *N*, *N*′-methylene-bis (acrylamide) as a cross-linking agent, and covalently immobilized PGA on the surface of paramagnetic polymer microspheres by reacting the amino group of PGA molecules with the epoxy group of paramagnetic polymer microspheres. The results showed that the initial activity of PGA immobilized on paramagnetic polymer microspheres was as high as 430 U g^−1^ (wet), and 99% of the initial activity was maintained after 10 cycles of use. In addition, the immobilized PGA has high thermal and pH stability and good reusability, and can be quickly recycled with the help of magnets.

In addition, Liu *et al.*^115^ synthesized novel carriers *via* cerium-bound mesoporous structured foam (Ce-MCF) to immobilize PGA *via* Lewis acid–base interaction (Fig. 11b). Due to the binding to Ce, PGA was firmly immobilized on Ce-MCF by interacting with the surface-stabilized Lewis acid sites. It was also shown that Ce-MCF support material was superior to Si-MCF in terms of operational stability, and after 10 cycles, PGA/Ce-MCF retained 90% of its main enzyme activity, which was much higher than that of PGA/Si-MCF (77%). Wu *et al.*^116^ used a reversed-phase microemulsion method to prepare Fe_3_O_4_ nanoparticles (NPs), and then coated the Fe_3_O_4_ NPs with β-cyclodextrin (β-CD) in the process of doping Mg^2+^. Then glutaraldehyde (GA) was grafted onto the surface of the material by acetal reaction to obtain Mg^2+^-Fe_3_O_4_@β-CD-g-GA NPs, and the covalent bond immobilization of PGA was achieved by using the Schiff base reaction of the aldehyde group with the PGA amino group (Fig. 11c). It was shown that the immobilized PGA had better operational and storage stability. After 11 repetitions, the immobilized PGA still had 56% of the initial viability and the carrier recovery was 86.6%.

##### 3.2.1.2 Hydroxyapatite

**Fig. 12:**
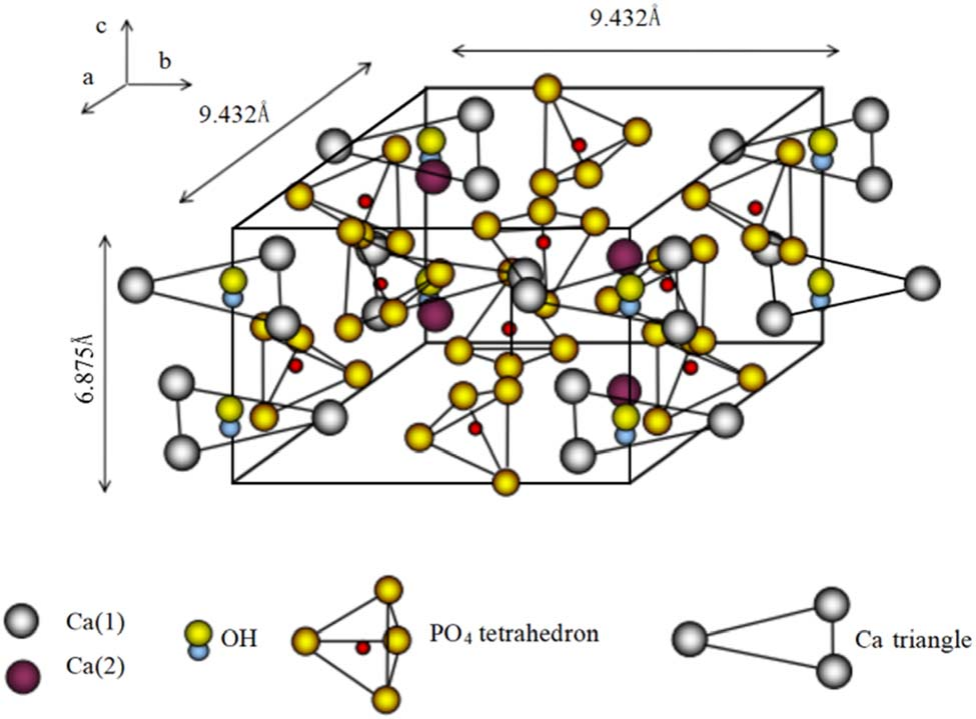
Schematic diagram of the crystal structure of hydroxyapatite [Adapted with permission from ref. 117 Copyright 2002 Chinese Academy of Medical Sciences. Permission required].

Hydroxyapatite (HA, Ca_10_(PO_4_)_6_(OH)_2_) is considered a calcium–phosphorus type bioceramic material widely used in many health applications, which is composed of calcium ions, tetrahedrally arranged phosphate groups and hydroxyl groups, forming a hexagonal crystal structure^117^ (Fig. 12). Its important property is the ability to dope various ions (cations such as Sr^2+^, Mg^2+^, Cd^2+^, Mn^2+^, Pb^4+^, Cr^3+^, Na^+^, K ^+^, Li^+^, *etc.*; anions such as CO_3_^2−^, SO_3_^2−^, AsO_4_^3−^, VO_4_^3−^, F^−^, Cl^−^, *etc.*) as needed, thus changing the physical, chemical and biological properties of HA.

HA has important applications due to its high adsorption capacity, biocompatibility, bioactivity, non-toxic nature, molecular affinity and catalytic properties. It has been successfully applied in biopharmaceuticals, sensors, the polymer industry, agriculture, catalysis and other fields. Due to its component characteristics, its positively charged oxygen ion and charged calcium ion (Ca^2+^) associated with phosphate anion (PO_4_^3−^) can interrelate electrostatically with the side groups of proteins and have the ability to bind to proteins to a certain extent, so HA has been widely studied as an enzyme immobilization carrier in recent years. Coutinho *et al.*^118^ effectively immobilized β-glucosidase by a very simple adsorption method on HA nanoparticles with enzyme activity recovery of up to 90% and good temperature and pH stability, and the immobilized β-glucosidase maintained about 70% of its initial activity even after 10 times repeated use. In addition, when HA is modified with metal ions such as Cu^2+^, Zn^2+^, Ni^2+^ and Co^2+^, the metal groups in HA particles can complex with the carboxylic acid group (COO^−^) of the enzyme, resulting in a very stable interaction, which makes it even more potential as an enzyme substrate. However, although HA is an inorganic carrier, its low strength, poor toughness and brittleness and lack of mechanical strength limit its development as an immobilization carrier to some extent.

Immediately after that, Almulaiky *et al.*^119^ modified HA using ZrO_2_ to enhance the carrier's mechanical and surface properties and immobilize α-amylase. The results showed that HA-ZrO_2_ had better environmental tolerance than free enzyme and HA immobilized α-amylase. In addition, there are many reports on the composite of HA with magnetic materials, which can not only enhance the mechanical properties of HA by using magnetic materials but also solve the recovery problem of biocatalysts simultaneously. The immobilization of xylanase phytase, and β-glucosidase, was made possible by Coutinho *et al.*,^120^ who use of the co-precipitation approach to creating HA/CoFe_2_O_4_ composites with various HA to CoFe_2_O_4_ mass ratios as carriers for immobilized enzymes.

#### 3.2.2 Organic carrier

Organic carriers have a looser architecture than inorganic carriers, which reduces their mass transfer resistance and speeds up their response time. The surface is rich in functional groups, and it is simple to modify the microenvironment of the carrier to achieve PGA immobilization. In addition, its biocompatibility is favorable to enhance the PGA enzyme activity. According to its source, it can be divided into natural organic carriers and synthetic organic carriers. Natural polymers such as cellulose, starch, agarose, cross-linked dextran, *k*-carrageenan, chitosan, gelatin, and collagen are widely utilized as carriers for immobilized enzymes albumin, sericin, cotton fibers, and liposomes. Natural polymers have properties and advantages such as biocompatibility, degradability, non-toxicity, physiological inertness, antibacterial properties, heavy metal chelating properties, easy gel formation, wide source, non-contamination, renewable, hydrophilic and amazing affinity to proteins. Due to their qualities and benefits, these materials are widely used in many industries, including as carriers for enzyme immobilization.

##### 3.2.2.1 Cyclodextrins

**Fig. 13:**
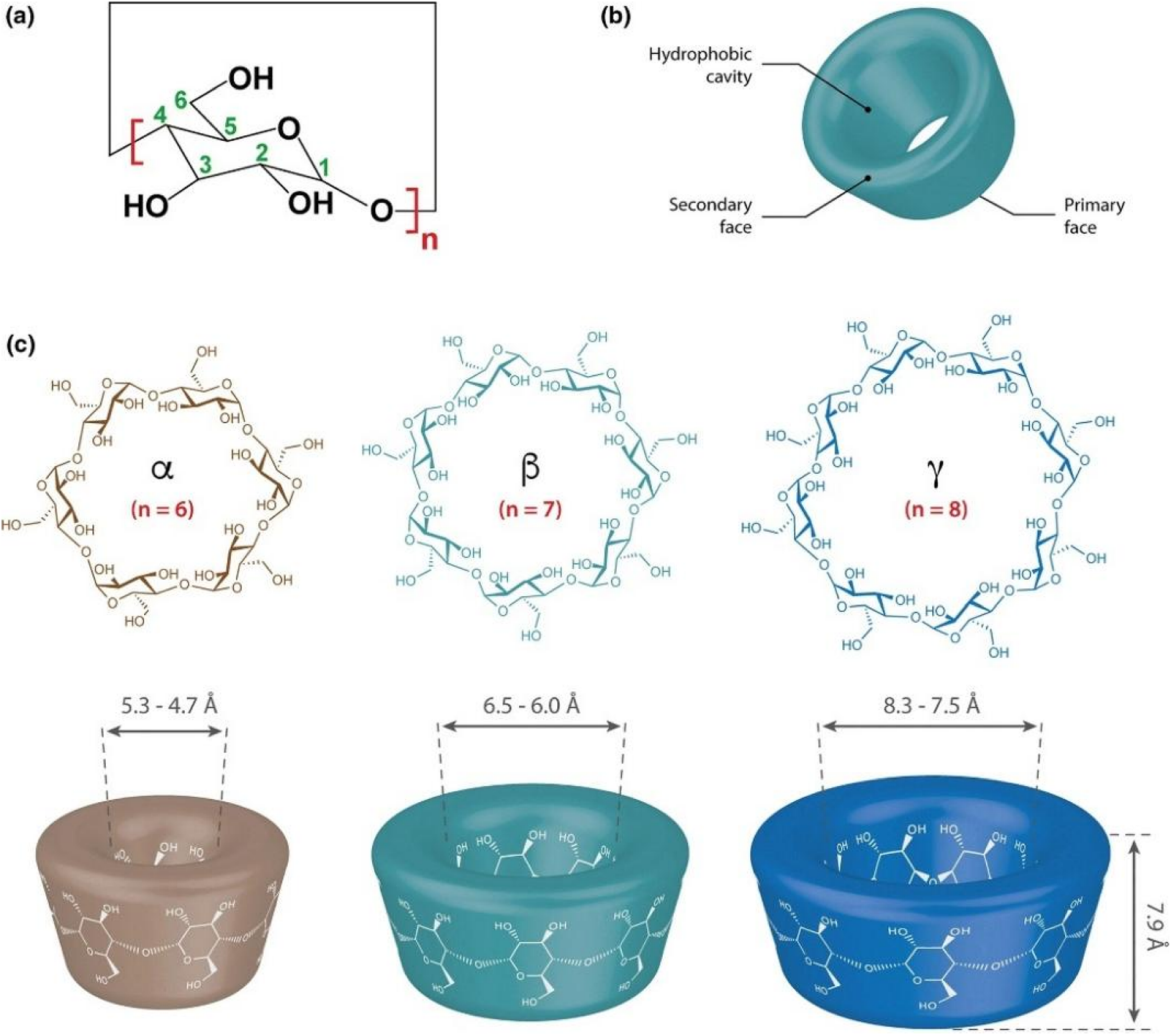
Schematic representation of the chemical structure of cyclodextrins; (a) the molecular structure formula of cyclodextrin; (b) schematic representation of the three-dimensional structure of cyclodextrins; (c) chemical structure and dimensions of α-, β- and γ-cyclodextrins (*n* = 6, 7 and 8) [Adapted with permission from ref. 122 Copyright 2018 Springer Nature. License number: 6243511336332].

Cyclodextrins (CDs), also known as cyclic straight-chain starch, cyclomaltose and chardonnay, are produced by the intramolecular transglycosylation reaction of starch degraded by cyclodextrin glucosyltransferase (CGTase).^121^ CDs is a more widely used natural carrier for PGA immobilization, it is a cyclic oligosaccharide composed of 6 (α-cyclodextrin/α-CD), 7 (β-cyclodextrin/β-CD), 8 (γ-cyclodextrinγ-CD) or more d-glucopyranose units linked by α-(1,4) bonds^122^ (Fig. 13), a hollow, truncated conical molecule with several glucose units covalently linked together by oxygen atoms and hydrogen bonds between secondary hydroxyl groups maintain the shape on adjacent units at the wider edges of the cavity. Because of its affordability, non-toxicity, odorlessness, and capacity to form complexes for various chemicals, β-CD is one of the most studied and often utilized. The ability of the β-CD molecule to form inclusion compounds with other substances through host–guest interactions is, in fact, similar to other forms of cyclodextrins. Seven glucose units and 21 hydroxyl groups make up its center cavity, which has a unique structure with outside hydrophilic and inner hydrophobic properties. The ability of the structure's core to trap or encapsulate other substances can enhance the guest molecule's physical, chemical, and biological characteristics.

On the basis of the unique cavity structure of CD, which is “hydrophilic on the outside and hydrophobic on the inside”, it is not only possible to make use of its active –OH to modify it functionally according to specific needs but also to make full use of the hydrophobic cavity structure to effectively encapsulate various compounds, such as specific small molecules, ions, and proteins. Moreover, this environmentally friendly cyclic oligosaccharide has the advantages of low toxicity, no contamination, and biocompatibility simultaneously.^92^ Therefore, it is widely used in drug transport, gene transport, catalytic reactions, separation, *etc.* and has laid an important foundation for researching and developing new materials. Among them, the application of CD in enzyme immobilization is also common.

**Fig. 14:**
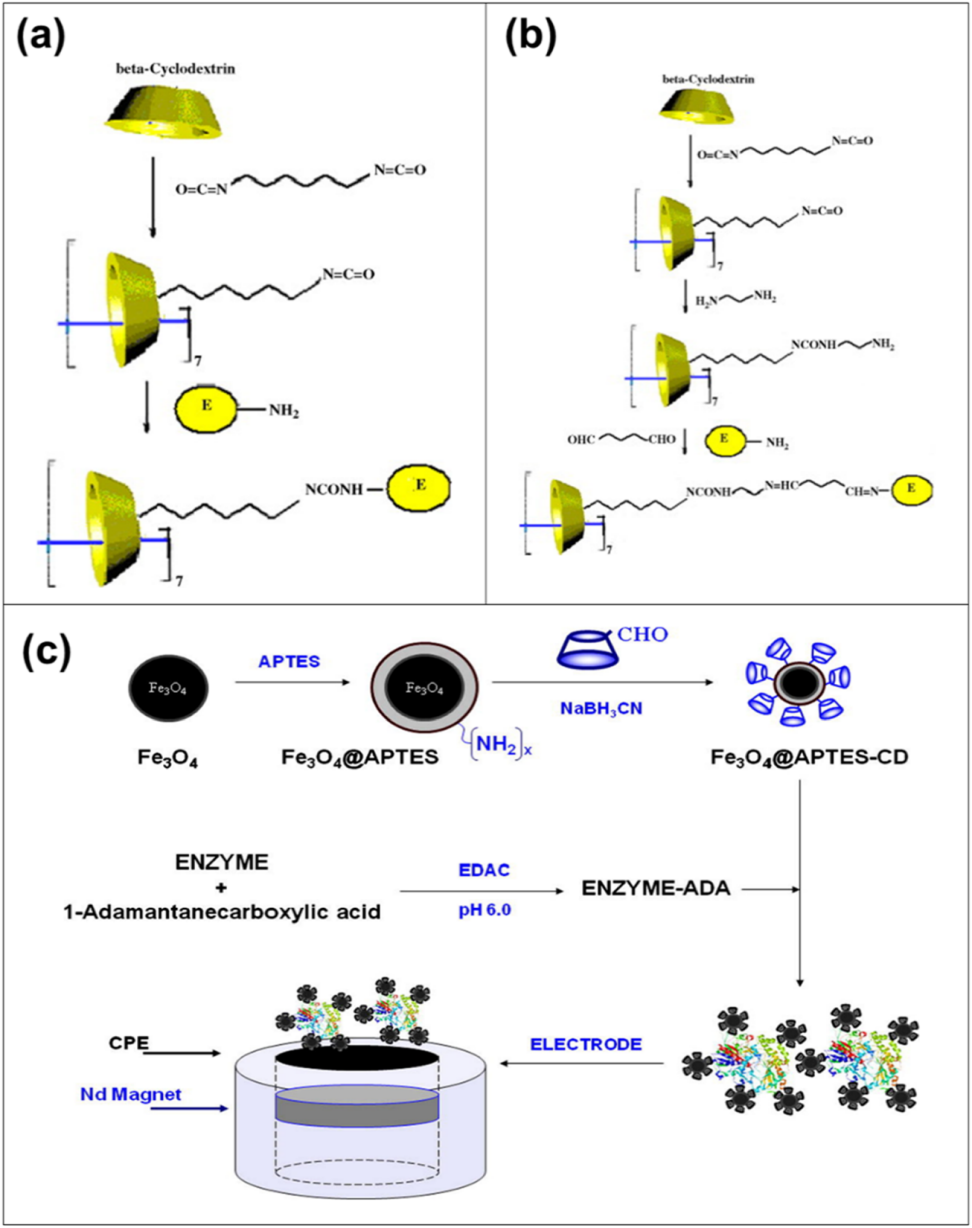
Schematic diagrams of the synthesis of βCD-based polymers and lipase immobilization methods: (a) hexamethylene diisocyanate cross-linking method; (b) glutaraldehyde cross-linking method [Adapted with permission from ref. 123 Copyright 2009 Elsevier. License number: 6243511474847]. (c) Schematic representation of the preparation of an enzyme biosensor from Fe_3_O_4_@APTES-CD magnetic nanomaterials [Adapted with permission from ref. 73 Copyright 2012 Elsevier. License number: 6243520096202].

For the immobilization of Candida lipase, Ozmen *et al.*^123^ synthesized cross-linked cyclodextrin polymers using hexamethylene diisocyanate (HMDI) and glutaraldehyde (GA) (Fig. 14a and b), and after optimizing the immobilization process, the immobilized lipase was physiochemically characterized. The outcomes demonstrated the superiority of the immobilized enzyme over the free enzyme. The initial activity of the immobilized lipase treated with HMDI and GA was maintained at about 56% and 82% after heat treatment at 60 °C for 120 min, and the activity of the immobilized lipase with the addition of GA was 62.75 U mg^−1^, which was 28.13 times higher than that of the immobilized lipase with HMDI; Dhiman *et al.*^56^ then immobilized partially purified β-mannanase on a sodium alginate modified substrate grafted β-CD and achieved 91.5% recovery of enzyme activity, pH and temperature stability were improved. The activity of the immobilized mannanase could be reused 15 times while remaining at 70%, and after 30 days of preservation at 4 °C, the activity dropped to 60%.

In addition, some researchers have also utilized composites composed of CD and enzymes for secondary utilization in the field of sensors, Díez *et al.*^73^ successfully prepared composites Fe_3_O_4_@APTES-CD by combining aldehyde-functionalized β-CD derivatives with superparamagnetic Fe_3_O_4_ nanoparticles coated with (3-aminopropyl) triethoxy silane through reductive alkylation with NaBH_3_CN, this use of oligosaccharide encapsulated core–shell nanoparticles as carriers to immobilize tyrosinase and xanthine oxidase through host–guest interactions, followed by modification of carbon paste electrodes to construct amperometric biosensors targeting catechol and xanthine (Fig. 14c).

##### 3.2.2.2 Polymer

Organic synthetic carriers possess mechanical strength and higher stability. Mechanosynthetic carriers are also well suited for a wide range of enzyme procedures because they make tuning the carrier target and microenvironment easier during synthetic preparation.^19^ Additionally, they are not more or less vulnerable to microbial infection harming them.

**Fig. 15:**
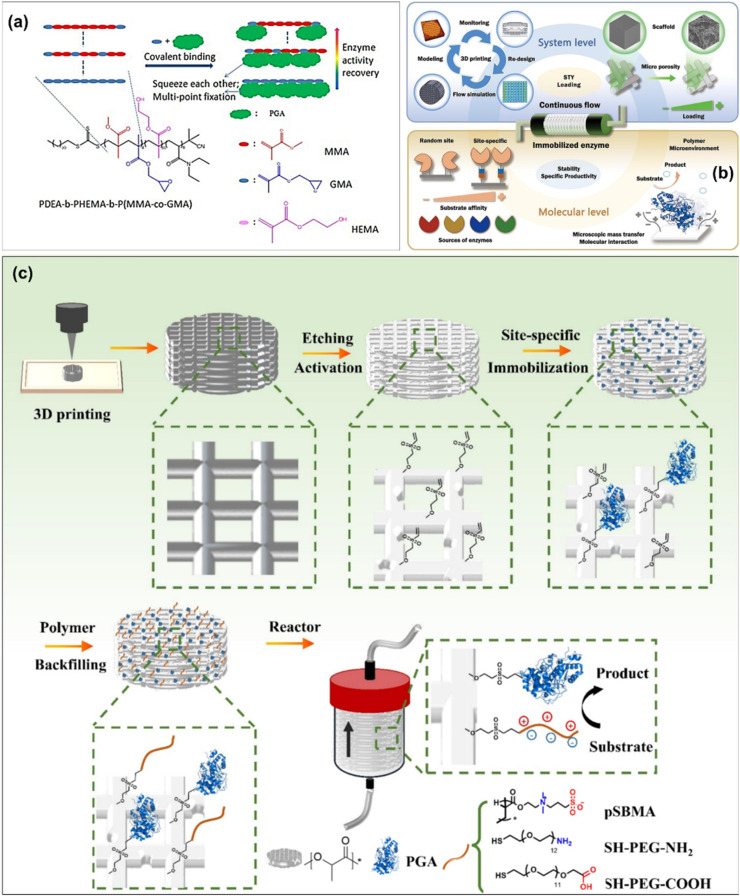
(a) Target spacing of thermo-sensitive carrier (MMA-co-GMA) on the activity recovery of immobilized PGA [Adapted with permission from ref. 109 Copyright 2019 Elsevier. License number: 6243520215192]. (b) Preparation of immobilized enzyme-filled bed reactor from 3D-printed polylactic acid scaffolds. (c) Chemical modification of three-dimensionally printed scaffolds for enzyme immobilization [Adapted with permission from ref. 127 Copyright 2025 Elsevier. Permission required].

Polypropylene, polyamides, polyvinyl alcohol, polyacrylates, polystyrene, nylon, and copolymers of ethylene and maleic anhydride or polypeptides, polyalkaldehydes, and styrene are organic synthetic polymers used as carriers. Li *et al.*^124^ prepared immobilized PGA carriers using reactive monomer acrylic acid and cross-linking agent divinylbenzene; Xue *et al.*^125^ used the reactive epoxy group of glycidyl methacrylate (GMA) as the target site for immobilized PGA, constructed hydrophilic microenvironment using *N*-vinylpyrrolidone (NVP). *N*, *N*′-methylene bisacrylamide (MBAA) as the findings revealed that the immobilized PGA had good operational stability (pH, temperature) and storage stability and remained at 96% of the initial viability after 15 times of repeated use. In addition, smart (photosensitive, heat-sensitive, pH-sensitive, *etc.*) polymers have been developed for the preparation of immobilized PGA carriers under the unremitting efforts of researchers. Tu *et al.*^126^ synthesized a class of thermosensitive block resins, PDEA-b-, using a reversible addition-break chain transfer reaction with *N*, *N*-diethylacrylamide (DEA), poly(β-hydroxyethyl methacrylate) (PHEMA) as a hydrophilic block, and poly(glycidyl methacrylate) (PGMA) as a hydrophobic block (and as a target for immobilization of PGA). PHEMA-b-PGMA (DHGs) to immobilize PGA, which significantly enhanced the activity and stability of the enzyme. Li *et al.*^109^ utilized glycidyl methacrylate (GMA) was used as the immobilization target and methyl methacrylate (MMA) was used as the copolymer monomer to rapidly and efficiently synthesize thermosensitive copolymers with different target spacings *via* a reversible addition reaction, PDEA-b-PHEMA-bP (MMA-co-GMA). And when the molar ratio of MMA to GMA in the copolymer was 8.75 : 1, the activity recovery of immobilized PGA was up to 63.50% (Fig. 15a).

Dong *et al.*^127^ printed PLA scaffolds using 3D printing and obtained microporous scaffolds with high specific surface area by an etch-activation process due to the construction of immobilized enzyme-filled bed reactor (Fig. 15b). It was shown that the microporous PLA scaffolds with the ability to regulate enzyme orientation and microenvironment prepared by developing an etch-activation method had a significant positive effect on the immobilization of PGA (Fig. 15c).

**Fig. 16:**
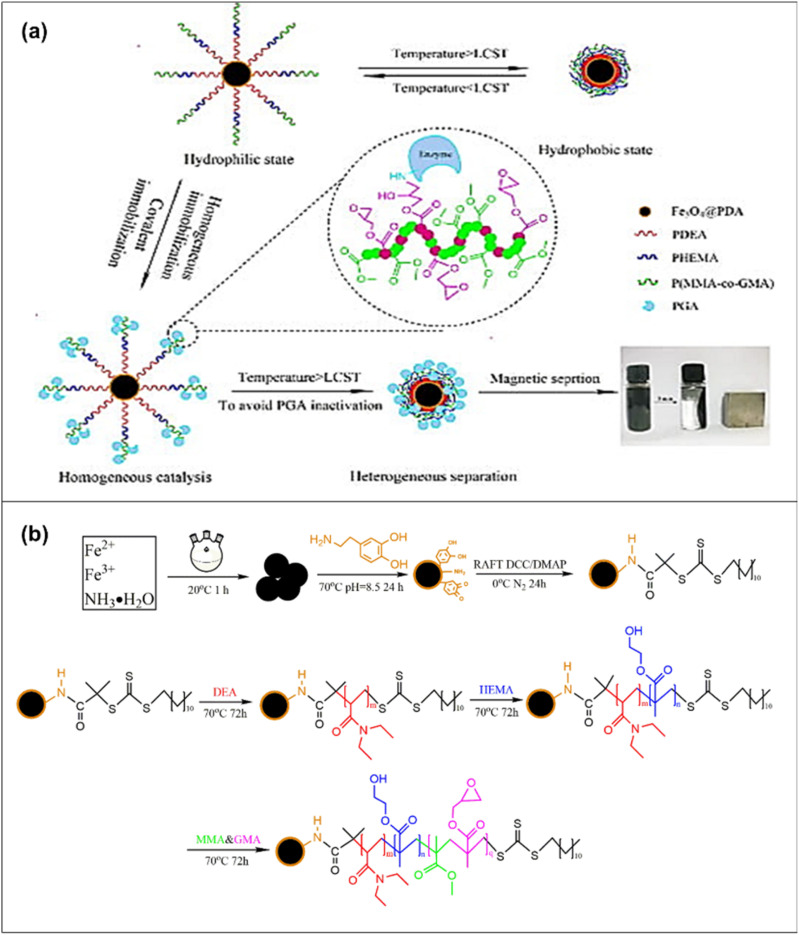
(a) Preparation of magnetic temperature-sensitive polymer composite carrier (Fe_3_O_4_@PDA-g-PDEA-b-PHEMA-b) and study on immobilized PGA [Adapted with permission from ref. 128 Copyright 2021 World Scientific Publishing Company. License number: 600251318].

In addition, composite carriers constructed from inorganic and organic carriers are also commonly used as immobilization carriers for PGA. As shown in Fig. 16a, Tu *et al.*^128^ used dopamine (DA) to form a poly(dopamine) (PDA) coating on the surface of Fe_3_O_4_ NPs. Then, the obtained Fe_3_O_4_@PDA NPs were modified with reversible addition breakage chain transfer (RAFT) reagent containing carboxyl groups on the surface, and the magnetic temperature-sensitive composite carriers Fe_3_O_4_@PDA-g-PDEA-b-PHEMA-b were prepared by the “Grafting from” strategy and RAFT polymerization method. PHEMA-b-P(MMA-co-GMA) NPs (Fig. 16b). The findings revealed that the relative enzyme activity (*R*_t_) of the immobilized PGA stored for 90 days was 83.9%, and 90.3% of the initial enzyme activity was maintained after 11 repeated uses.

##### 3.2.2.4 Porous organic frameworks (POFs)

POFs, including metal organic frameworks (MOFs), covalent organic frameworks (COFs) and hydrogen-bonded organic frameworks (HOFs), are next-generation porous materials with diverse topologies. POFs overcome the limitations of traditional porous materials, such as structural rigidity and amorphousness, by offering ultrahigh surface areas, tunable porosity, and robust topologies. These features enable efficient enzyme immobilization *via* surface grafting or cavity internalization, ensuring high loading capacity while preserving enzyme conformation and activity through protective confinement that permits substrate/product diffusion. The versatile synthesis of POFs supports diverse immobilization strategies, including biomineralization and coprecipitation, while their functionalized components can confer enzyme-like activity, facilitating novel cascade reactions. These characteristics make POFs materials highly promising for the immobilization of PGA.^129^

**Fig. 17:**
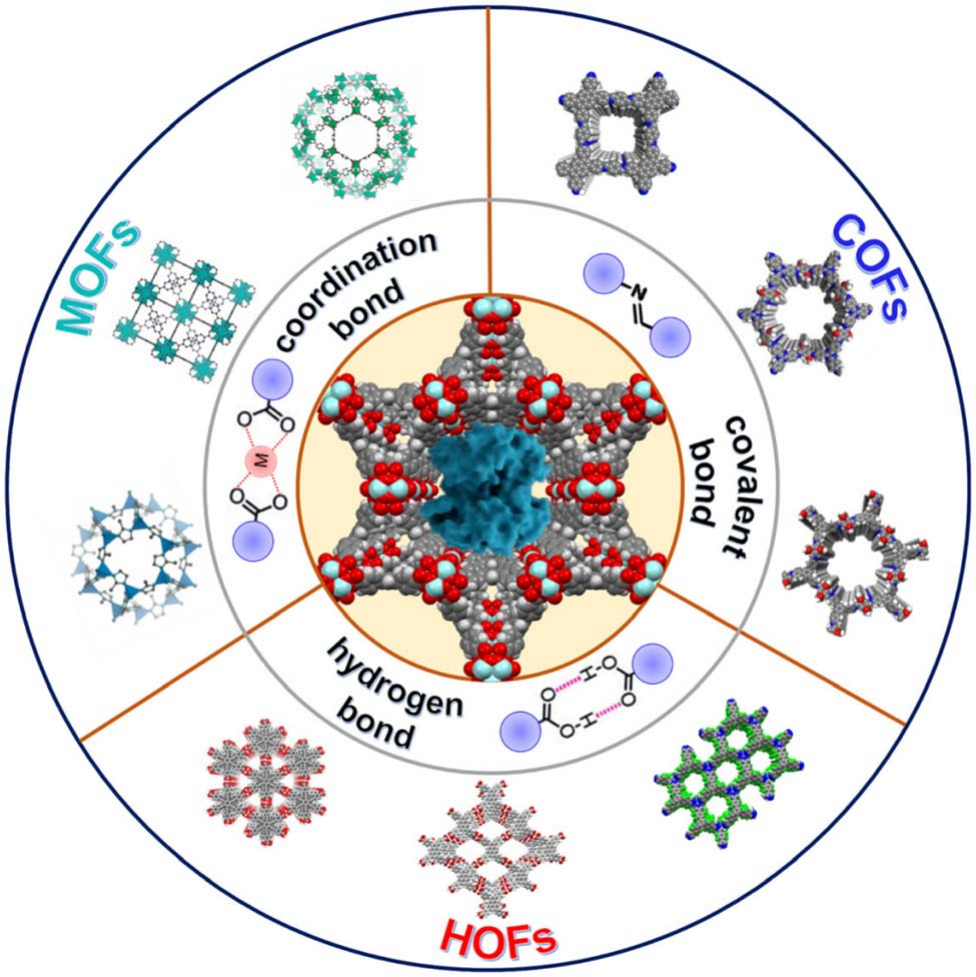
The structures of porous organic frameworks (including MOFS, COFS, and HOFS) [dapted with permission from ref. 129 Copyright 2022 Royal Society of Chemistry. License number: 1716831-2].

**Fig. 18:**
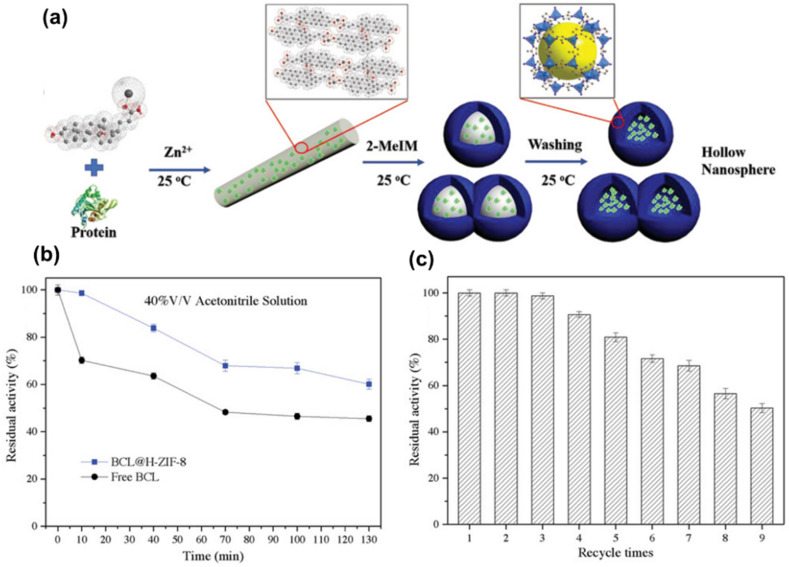
(a) Schematic diagram of the synthesis process of hollow composite spheres based on metal–organic frameworks induced by proteins. (b) Comparison of the stability of free proteins and immobilized proteins. (c) Reusability of ZIF8 as an enzyme immobilizer [Adapted from ref. 131 which is an open-access publication licensed under CC-BY 4.0].

MOFs are formed through self-assembly of metal nodes and organic ligands into highly ordered crystalline porous structures (Fig. 17). The pore size of these structures can be adjusted (typically being micropores, and some can reach mesopore range), and the specific surface area is high (>1000 m^2^ g^−1^).^130^ Du *et al.* established a controllable and simple strategy for constructing hollow composite materials based on MOFs through a protein-induced soft template approach. Using PGA as the soft template, PGA/MOF composite materials were fabricated, and this composite material exhibited universality among ZIF-8, ZIF-67, and Fe-MOF materials. The synthesis process is shown in Fig. 18a. As shown in Fig. 18b and c, the study demonstrated that this composite material exhibited high biological activity and stability.^131^ When using MOF as the enzyme immobilization carrier, methods such as surface grafting immobilization, *in situ* encapsulation immobilization, and post-synthesis osmotic pressure immobilization are commonly employed. At present, there are relatively few studies on the immobilization of PGA using MOF materials, but these typical strategies have already demonstrated considerable application potential.^132^

COFs are formed by connecting organic monomers through covalent bonds to create a crystalline framework. They feature regular one-dimensional pores (with pore diameters ranging from 1.5 to 6.0 nm) and exhibit excellent thermal and chemical stability.^133^ COFs exhibit MOF-like structural advantages, including tunable porosity, high surface areas, and tailorable functionalities, while offering superior biosafety and stability due to their metal-free composition and robust covalent linkages. Their structural predictability enables precise design of pore architectures for targeted enzyme immobilization.^134^ In contrast, HOFs utilize dynamic non-covalent interactions (*e.g.*, hydrogen bonding, π–π stacking) for assembly, resulting in lower inherent stability than COFs or MOFs.

Despite the significant potential of POFs in enzyme immobilization, their application for PGA fixation remains underexplored compared to other enzyme systems. Key limitations include the inherent trade-off between enzyme stability and mass transfer efficiency, the confined porous architecture often restricts substrate diffusion and product transport, thereby reducing catalytic kinetics. Furthermore, the fundamental bio–interface interactions between PGA and framework hosts (MOFs, COFs, or HOFs) are poorly understood at the molecular level, necessitating advanced characterization techniques such as *in situ* spectroscopy or cryo-electron microscopy to elucidate structure–activity relationships. Thus, despite theoretical promise, experimental studies on POF-based PGA carriers are still nascent and require concerted efforts in material design and interfacial engineering to bridge the gap between laboratory innovation and industrial implementation.

#### 3.2.3 Organic–inorganic composite carriers

Organic–inorganic composite carriers play a significant role in the immobilization of PGA. As previously discussed in separate sections regarding inorganic and organic carriers, composite carriers effectively combine the advantages of both material types. They exhibit the high functional-group reactivity and ease of modification characteristic of organic components, along with the exceptional stability and facile separability offered by inorganic supports. Owing to this synergistic combination, organic–inorganic hybrid carriers have become the most widely utilized system for immobilization applications. Therefore, a detailed elaboration will not be reiterated here.

### 3.3 Problems of immobilized PGA

Despite the enhanced reusability and operational stability of immobilized PGAs compared to their free counterparts, the recovery of enzymatic activity remains a significant challenge. This is primarily attributed to a loss of active site integrity resulting from factors such as damage during the reaction process, an improperly designed microenvironment, chemical bonding of the immobilized PGA, and steric hindrance between PGA molecules and the carrier. To address this, the development of immobilized PGAs with both high enzymatic activity and robust functional properties, including reusability, is crucial. According to relevant reports from domestic and international studies,^135^ Mn and Mg have positive effects on biomolecules (enzymes, cells, *etc.*), and Mn^2+^ and Mg^2+^ have been frequently reported to enhance the catalytic activity of enzymes. It has even been reported that Mn^2+^ and Mg^2+^ can form coordination cross-linking bonds with enzymes with certain strengths.

**Fig. 19:**
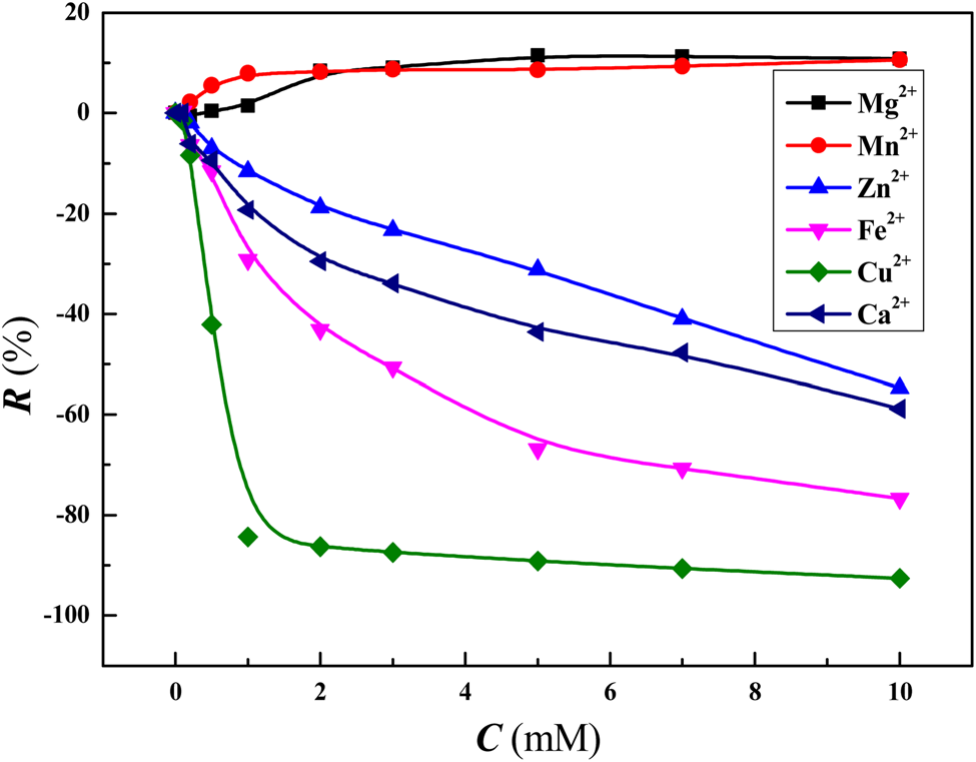
Relationship between the concentration of divalent metal ions and the rate of change of free PGA viability.

Meanwhile, our experimental results also proved the enhancement effect of Mn^2+^ and Mg^2+^ on the catalytic activity of PGA (the relationship between the concentration of divalent metal ions and the rate of change of free PGA activity is shown in Fig. 19, where *R* (%) represents the growth rate of free PGA enzyme activity; *C* (mM) represents the concentration of divalent metal ions, and all of them used chloride (MgCl_2_, MnCl_2_, ZnCl_2_, FeCl_2_, CuCl_2_, CaCl_2_) for metal solution preparation), based on which doping by Mn^2+^, Mg^2+^ was carried out during the design of the carrier microenvironment to stabilize the conformation of PGA to a certain extent during immobilization. To improve the effect on the immobilized PGA enzyme activity, both may simultaneously operate as a link crossing the enzyme to the substrate.^136^ The appropriate carrier should have a high enzyme loading capacity, a quick response time, and a high recovery rate in addition to the microenvironment. Unlike catalytic activity, the three markers mentioned above are partly bound by one another. For instance, a greater specific surface area of the carrier is needed to improve the enzyme loading of immobilized carriers; this can be done by either utilizing porous carriers or lowering the carrier's particle size. The immobilization function will be further weakened by too small of particle size, resulting in a reduced recovery and more challenging separation.^71^ On the other hand, excessive spatial site resistance can negatively impact the response rate if PGA is immobilized on a porous carrier. Therefore, the focus of attention is the question concerning how to design and produce an optimal carrier with a high recovery rate, quick reaction rate, high loading capacity, and catalytic activity.

### 3.4 Effect of immobilization on enzyme performance

#### 3.4.1 Ionic strength

The effect of ionic strength is a key factor in the study of the immobilization of PGA. In 2022, Rocha *et al.*^137^ explored the optimization of stability and activity under specific conditions by immobilizing PGA on vinylsulfone-agarose (VS-agarose). It was found that the ionic strength had a significant effect on the immobilization of PGA, and the appropriate ionic strength could improve the stability and catalytic efficiency of the enzyme. This finding provides an important reference for further optimizing the immobilization conditions of PGA.

#### 3.4.2 Carrier microenvironment modulation

In terms of carrier microenvironment regulation, in 2013, Paolo Bonomi investigated the effect of immobilizing PGA on epoxy-activated acrylic carriers and found that the synthesis/hydrolysis ratio of the enzyme could be significantly improved by insertion of a spacer and bursting of nucleophilic reagents.^138^ In addition, in 2021, a study by Liu Chunli further investigated the effects of different modification methods on PGA immobilization on modified TiO_2_ carriers, and the results showed that these modification methods could effectively enhance the performance of the enzyme.^139^ These studies showed that the catalytic efficiency and stability of PGA could be significantly improved by optimizing the carrier microenvironment.

#### 3.4.3 Operational stability after immobilization

In terms of operational stability after immobilization of PGA, recent studies have proposed various methods to improve its stability and recovery efficiency. In 2014, Yang *et al.* studied paramagnetic epoxy-functionalized mesocellular foams (PEMCFs) with an open pore system and found that this material was able to support highly active and stable immobilization of PGA and could be magnetically fielded for easily recycled.^140^ Also in 2014, Yang developed a paramagnetic Fe_3_O_4_ nanocomposite, which not only supports high initial activity and stability of PGA, but also can be easily recycled by a magnetic field.^141^ In addition, Zhan *et al.* in 2014 improved the immobilization stability of PGA by epoxidation functionalization of cubic Ia_3_d mesoporous silica, but the activity decreased due to reduced pore size and increased hydrophobicity.^142^ These studies show that optimizing the immobilization process of PGA by different materials and methods can significantly improve its stability and operational performance in industrial applications.

### 3.5 High-throughput screening and analytical methods

High-throughput detection and analysis represent an advanced approach to research that involves the rapid, automated evaluation of numerous variables and conditions. This methodology has a transformative effect on immobilized enzyme technology by accelerating the entire development life cycle. It enables the efficient discovery of ideal environmental parameters for cultivating microbes to maximize enzyme yield and facilitates the identification of compounds that could inhibit or enhance enzyme activity. Furthermore, sophisticated digital frameworks and *in silico* simulations are used to predict optimal process parameters, such as the most suitable carrier materials and bonding methods, thereby refining the immobilization process itself. These methods also allow for a deeper understanding of the fundamental catalytic mechanisms of enzymes and their interactions with their carriers. The collective result is the creation of more robust, stable, and cost-effective immobilized enzyme systems with significantly improved catalytic performance.

#### 3.5.1 Computational simulation and bioinformatics tools

Enzyme immobilization is an important means to enhance enzyme stability and enable repeated use. The design of traditional PGA immobilization systems is highly complex and heavily relies on empirical trial and error. It faces numerous challenges such as large experimental volumes, difficulty in understanding the key limiting factors within the carrier, and the random fixation that can easily lead to the blocking of active sites. Furthermore, the high cost of the carrier preparation makes it particularly necessary to conduct rational optimization of the immobilization process. Unfortunately, relatively little work has been done in the field of rational design of protein immobilization, and most of these procedures focus on the rational design of immobilized biocatalysts.^143^

In recent years, computational simulation methods have played an increasingly important role in the development and mechanism research of the immobilization process of PGA. At the molecular level, molecular docking software (such as AutoDock, Discovery Studio) can be used to predict the binding sites, orientations, and interaction affinities between PGA and various functionalized carrier materials (such as magnetic composite carriers, chitosan), providing a theoretical basis for rational design of carrier surface modification strategies. On this basis, molecular dynamics simulations (such as GROMACS, NAMD) analyze the evolution trajectories of the immobilized enzyme conformation under external environmental disturbances (such as temperature, pH), revealing the molecular mechanisms of stability and enhanced tolerance at the atomic scale. Further, to optimize the reaction process in practical applications, enzyme kinetic modeling tools (such as COMSOL Multiphysics, MATLAB) can be used to integrate the experimentally determined enzymatic parameters (such as *K*_m_, *v*_max_) to construct a mathematical model of the catalytic reaction, for predicting and optimizing the key operating conditions in industrial reactors, significantly improving the rationality and efficiency of process development.

Taking into account the advantages of the above computational simulation techniques in optimizing the immobilization design of PGA, some recent studies have focused on building software platforms to achieve the rational design of PGA immobilized enzymes and the high-throughput prediction of their performance. Castillo *et al.* systematically introduced and applied a computational strategy named RDID (Rational Design of Immobilized Derivatives) and its accompanying software RDID_10_. They used mathematical algorithms and bioinformatics tools to predict and optimize the immobilization process of PGA on aldehyde-activated carriers. Mainly, they efficiently predicted immobilization conditions, configurations, activity and stability at the computational level, significantly reducing the amount of experimental work. Moreover, it is applicable to various enzymes and carrier systems, and supports high-throughput screening and rational design. Computational modeling has been extensively employed to optimize PGA production and immobilization processes.^144^ In 2013, Premalatha *et al.* proposed a hybrid model integrating artificial neural networks (ANN) and ant colony optimization (ACO), which significantly enhanced the activity of recombinant PGA in *E. coli* compared to conventional methods.^145^ Concurrently, Wei *et al.*^146^ utilized quantum mechanics/molecular mechanics (QM/MM) simulations to elucidate the catalytic cycle mechanism of *E. coli*-derived PGA, despite its widespread industrial use, the detailed enzymatic mechanism remains partially unresolved. In immobilization research, Chen *et al.*^147^ evaluated glutaraldehyde modified TiO_2_ as a carrier for PGA immobilization. Computational modeling was applied to determine optimal immobilization parameters, resulting in improved catalytic performance. These studies underscore the pivotal role of computational approaches in refining PGA production and immobilization strategies, offering theoretical frameworks for enhancing efficiency and product quality.

#### 3.5.2 High-throughput screening techniques

In the field of high-throughput screening for PGA, Teresa investigated multiple factors influencing PGA production during fed-batch cultivation of recombinant *Escherichia coli* (r*E. coli*), including culture media, cultivation strategies, and temperature. The study identified 20 °C as the optimal temperature for maximizing PGA yield.^42^ In 2020, Li *et al.*^72^ developed a fluorescent probe (PNA) for detecting PGA in bacterial systems and conducted high-throughput screening of natural inhibitors. Their results demonstrated that oleanolic acid effectively inhibits PGA activity. These advancements provide critical technical support for optimizing PGA production conditions and screening potent inhibitors.

#### 3.5.3 Fluorescent probe detection

For PGA detection, Tian *et al.* introduced a novel near-infrared fluorescent probe (DDAP) capable of detecting and visualizing endogenous PGA activity in bacterial cells, thereby facilitating its functional analysis and biotechnological applications.^148^

## 4. Conclusion and perspectives

In this review, the sources, structure, catalytic mechanism, immobilization strategies, carrier materials, and industrial applications of PGA have been systematically summarized. Immobilization has been demonstrated to significantly improve enzyme stability, reusability, and operational robustness, making it highly suitable for industrial applications. However, despite these advances, several limitations remain. In many cases, immobilization leads to a reduction in catalytic efficiency, which is closely associated with kinetic limitations such as mass transfer resistance and diffusion constraints. At the same time, the enhanced stability of immobilized PGA originates from thermodynamic effects, including conformational stabilization and microenvironmental regulation. Therefore, the overall performance of immobilized PGA reflects a balance between kinetic limitations and thermodynamic stabilization.

Current immobilization strategies, including adsorption, covalent binding, encapsulation, and cross-linking, each offer specific advantages but also present inherent limitations. No single approach can simultaneously achieve high activity, long-term stability, and efficient mass transfer, which restricts their industrial application. From an industrial perspective, further challenges remain in terms of scalability, cost-effectiveness, and long-term operational stability.

Future research should focus on the rational design of immobilized systems that balance kinetic and thermodynamic effects. Promising directions include:

(1) Hierarchical and porous carriers to reduce diffusion resistance: developing innovative immobilization strategies and microenvironments to prevent the loss of enzymatic activity recovery due to active site damage and steric hindrance.

(2) Dynamic or stimuli-responsive systems to regulate enzyme microenvironment: designing advanced carrier systems and optimizing processes to overcome the inherent mass transfer limitations that hinder catalytic efficiency in immobilized systems.

(3) Exploring safer microbial hosts: investigating and utilizing alternative, robust microbial strains that are Generally Recognized as Safe (GRAS), to reduce the reliance on *E. coli* for industrial production.

(4) Development of scalable and cost-effective materials for industrial applications.

Overall, future progress in immobilized PGA systems will depend on moving from empirical optimization toward mechanism-guided design, which is essential for achieving high-performance and industrially viable biocatalytic processes.

## Author contributions

All authors contributed to the study conception and design. Document indexing, data collection and analysis were performed by P. J., H. Y. T. and N. W. The first draft of the manuscript was written by P. J., Z. B. C., F. Y. L., and X. D. W. And all authors commented on previous versions of the manuscript. All authors read and approved the final manuscript.

## Conflicts of interest

The authors declare no competing interests.

## Data Availability

No primary research results, software or code have been included and no new data were generated or analysed as part of this review.
